# Adipose Stromal Cell-Derived Cancer-Associated Fibroblasts Promote Pancreatic Adenocarcinoma Progression Through SFRP4 Signaling

**DOI:** 10.3390/cancers18020233

**Published:** 2026-01-12

**Authors:** Joseph Rupert, Lingyi Cai, Alexes C. Daquinag, Dimitris Anastassiou, Mikhail G. Kolonin

**Affiliations:** 1 Center for Metabolic and Degenerative Diseases, Institute of Molecular Medicine for the Prevention of Disease, McGovern Medical School, The University of Texas Health Sciences Center at Houston, Houston, TX 77030, USA; joseph.e.rupert@uth.tmc.edu (J.R.); alexes.daquinag@uth.tmc.edu (A.C.D.); 2 Department of Systems Biology, Columbia University, New York, NY 10032, USA; lingyi.cai@columbia.edu; 3 Department of Electrical Engineering, Columbia University, New York, NY 10027, USA; 4 Herbert Irving Comprehensive Cancer Center, Columbia University, New York, NY 10032, USA

**Keywords:** cancer, tumor microenvironment, metastasis, pancreas, fibroblast, stromal, adipose, LINC01614, SFRP4, Wnt, TGF, SMAD

## Abstract

**Background/objectives:** Progression of pancreatic ductal adenocarcinoma (PDAC) and other carcinomas relies on cancer-associated fibroblasts (CAFs). A subset of CAFs is derived from adipose stromal cells (ASCs) recruited by tumors and the ASC-CAF conversion has been associated with invasiveness and poor prognosis. **Methods:** To explore the underlying molecular mechanisms, we used a model based on primary ASCs derived from human visceral adipose tissue co-cultured with human PDAC cell line Capan-1. To investigate cancer progression in vivo, we also used mice orthotopically grafted with mouse KPC cells. **Results:** Genomic analysis revealed that Capan-1 co-culture induces Wnt and TGFβ signaling and extracellular matrix (ECM) gene expression in ASC. We investigated the function of two markers of the fibroblastic transition highly induced by cancer cells: a long non-coding RNA *LINC01614* and a Wnt signaling modulator *SFRP4*. By using ASCs with either *SFRP4* or *LINC01614* knocked out (ko), we showed that both genes are required for Wnt/TGFβ signaling and ECM induction in ASCs by Capan-1. Analysis of changes in Capan-1 genes that rely on *LINC01614* and *SFRP4* expression in ASCs also identified the Wnt and TGF pathways. *SFRP4* ko in ASCs suppressed both migration and invasion of Capan-1 cells. We show that tumors in *SFRP4* ko mice have less desmoplasia, less epithelial dedifferentiation, reduced growth rate, and reduced progression to metastasis. **Conclusions:** We conclude that *SFRP4* promotes cancer progression in pancreatic cancer and is a promising therapeutic target.

## Simple Summary

Cancer-associated fibroblasts (CAFs) promote the progression of pancreatic ductal adenocarcinoma by remodeling the microenvironment toward tumor growth, invasion, and metastasis. A sub-population of CAFs originates from adipose stromal cells in adjacent fat tissue through mechanisms that are not well understood. Using co-cultures of human adipose stromal cells with pancreatic cancer cells and a mouse model of pancreatic cancer, we found that tumor cells induce Wnt and TGFβ signaling and extracellular matrix gene expression in adipose stromal cells. We discovered that two important genes, the long non-coding RNA LINC01614 and the Wnt signaling modulator SFRP4, are required for this transition. Loss of SFRP4 reduced cancer cell migration, growth, and metastasis, suggesting that SFRP4 is a promising therapeutic target inhibiting the transition.

## 1. Introduction

Despite the progress in cancer medicine, pancreatic ductal adenocarcinoma (PDAC) remains largely incurable. The majority of patients who present with disseminated disease are offered palliative chemotherapy. For certain subtypes of PDAC, chemotherapeutics can prolong survival, but recurrent resistance remains a challenge, with loss of adipose tissue and subsequent cachexia contributing to mortality [1]. The tumor microenvironment (TME) plays an important role in cancer progression to aggressive stages [2]. Cancer-associated fibroblasts (CAFs) are a diverse and dynamic population of non-malignant mesenchymal cells within the TME. CAFs modulate tumor vascularization, remodeling of the extracellular matrix (ECM), proliferation, epithelial–mesenchymal transition (EMT), resistance to chemotherapy, metastatic dissemination, and immunosuppression [3]. In recent years, much progress in understanding CAF heterogeneity and plasticity has been made [4]. However, the origins of distinct CAF subpopulations remain to be debated [5]. Mouse PDAC models have revealed that pancreatic stellate cells contribute little to CAFs [6]. Multiple lines of evidence indicate that CAFs with pro-metastatic properties may originate from other sources of mesenchymal stroma [7].

The increased aggressiveness of various carcinomas, including pancreatic, breast, and prostate cancers, has been linked to obesity and the expansion of adipose tissue [8,9]. Adipose tissue surrounding carcinomas has been identified as a source of CAFs, which becomes particularly important in the context of obesity [10,11]. Studies in mouse models have demonstrated that inflamed and fibrotic adipose tissue in obesity enhances cancer progression [12,13]. Adipose stromal cells (ASCs) consist of a diverse population, including pro-inflammatory and myofibroblastic progenitors [13]. Studies based on adipose transplantation and lineage tracing have revealed that ASCs proliferate in obesity, become mobilized, and migrate to tumors, contributing to poor cancer prognosis in obese patients [14,15]. Furthermore, ASCs infiltrating tumors from adjacent adipose depots have been identified as contributors to CAFs [16]. These adipose-derived CAFs are believed to play a multifaceted role in promoting cancer progression by producing the ECM supporting angiogenesis and metastasis [17,18,19]. They have also been implicated in inducing EMT and enhancing cancer aggressiveness [20,21,22]. However, the specific molecular mechanisms through which ASCs transition to CAFs promoting cancer progression have remained poorly understood.

The clinical relevance of these observations from mouse models has been corroborated by our analysis of single-cell RNA sequencing (RNA-seq) data from human PDAC, which has revealed a gene expression trajectory strongly suggesting that CAFs in aggressive tumors originate from ASCs that gradually evolve during disease progression [23]. Similar observations have been made by other groups in independent studies [24,25]. Based on computational genomic analysis, we have reported that high expression of *THBS2*, *INHBA*, and several collagens, with prominent presence of *COL11A1* marking the endpoint of the transition, is a signature of CAFs in aggressive tumors in multiple cancer types [23,26]. This gene co-expression is observed only in tumors that had progressed beyond a cancer type-specific stage threshold. For example, in ovarian cancer it appears only after having reached stage III (mainly omental metastasis), while in breast cancer it may appear as early as stage I, but never in carcinoma in situ, consistent with the interaction of cancer cells with ASCs. In this study, we refer to these “aggressive” CAFs as “aCAFs”. The reported computational analysis revealed the induction of genes coding for the secreted frizzled-related protein 4 (*SFRP4*) and the long intergenic non-protein coding RNA *LINC01614* (aka *LNC01614*, *AC093850.2*, *LCAL4*) in the ASC-aCAF transition [23]. The same analysis also identified a gene signature for the progenitor ASC population, which characterizes cells found among fibro-adipogenic progenitors. This population has both adipogenic and fibroblastic differentiation potential, the former being consistent with its presence in the stromal vascular fraction of adipose tissue and the latter with its transition to aCAFs following recruitment by cancer cells [27].

Here, we discover Hedgehog signaling [28] as a mechanism through which PDAC cells activate the ASC-aCAF transition. We identify *LINC01614* and *SFRP4* as the genes induced in human ASCs by co-culture with human PDAC cells. Our data demonstrate that *LINC01614* and *SFRP4* expression activate Wnt and TGFβ signaling linked with the production of ECM molecules, including COL11A1. We also show that SFRP4 secreted by ASCs promotes Wnt and TGF signaling and the resulting invasiveness of PDAC cells. SFRP4 is an evolutionary conserved secreted protein with a known mouse ortholog [29]. By using a mouse SFRP4-ko model, we demonstrate that pancreatic tumor growth, desmoplasia, and progression to metastasis are promoted by CAF-derived SFRP4.

## 2. Materials and Methods

### 2.1. Cells and Culture

Cells were cultured at 37 °C with 5% CO_2_. Human primary ASCs obtained from visceral adipose tissue of a healthy donor undergoing bariatric surgery and cryopreserved were reported previously [30]. Cells were expanded in DMEM with 20% fetal bovine serum (FBS) and stably transduced with a lentivirus pLVX-tdTomato-C1 (Takara Bio USA Inc., #632564; San Jose, CA, USA) expressing a red fluorescent protein, tdTomato red fluorescent protein (RFP), and carrying a puromycin selection gene. The RFP+ ASCs were subsequently transduced with a lentivirus Lenti-CRISPR v2-Blast (Addgene, #83480; Watertown, MA, USA) carrying the blasticidin selection gene and with guide RNA for CRISPR knockouts or without guide RNA (empty vector control). For LINC01614, a previously validated sequence CACCGGTGTAAGGTACTCAAGTGCT was used [31]. For SFRP4, a previously validated sequence ACCGACTTGCACGGCTTGAT was used. To select successfully transduced cells, cells were cultured in DMEM + 20% FBS+ 5 μg/mL blasticidin/puromycin for 7 days. Cells that were not transduced were also cultured in DMEM + 20% FBS+ 5 μg/mL blasticidin/puromycin alongside transduced cells to verify complete cell death with the selection antibodies. Stably transfected ko and control (empty RFP+ vector) cells were expanded upon blasticidin/puromycin selection. Capan-1 cells received from ATCC (HTB-79; Manassas, VA, USA) were expanded in DMEM with 20% FBS and stably transduced with a lentivirus pLVX-EGFP (Addgene, #128652) expressing enhanced green fluorescent protein (EGFP) and carrying a puromycin selection gene.

### 2.2. Gene Expression Analysis

Cells were trypsin-dissociated and co-cultured cells were subjected to FACS sorting with ARIA-II (BD Biosciences; Franklin Lakes, NJ, USA. Separated GFP+ and RFP+ cells were used for total RNA extraction as described previously [32]. For expression measurement by RT-PCR, mRNA was extracted using the Trizol Reagent (Life Technologies, #15596018; Carlsbad, CA, USA). Complementary DNAs were generated using High-Capacity cDNA Reverse Transcription Kit (Applied Biosystems, #4368814; Waltham, MA, USA). PCR reactions were performed on a CFX96 Real-Time System C1000 Touch thermal cycler (Bio-Rad; Hercules, CA, USA) using Q-PCR Master Mix (Gendepot, #Q5600-005, Altair, TX, USA). Expression of genes were normalized to *GAPDH* RNA. The Sybr green primers were as follows: *GAPDH* RNA Forward (CATCACTGCCACCCAGAAGACTG), Reverse (ATGCCAGTGAGCTTCCCGTTCAG); *SFRP4* Forward GCTTAGGCGTTTACAGTCAACATC, Reverse CTATGACCGTGGCGTGTGCATT; *LINC01614* Forward TCAACCAAGAGCGAAGCCAA, and Reverse TTGGACACAGACCCTAGCAC. Total RNA sequencing was performed by UTHealth Cancer Genomics Core. Principal component analysis (PCA) was used to evaluate inter-sample differences. Gene expression distribution was found to be comparable among all samples. To compare gene expression levels, the distribution of gene counts and expected number of fragments per kilobase of transcript sequence per million base pairs sequenced (FPKM) was assessed.

### 2.3. Bulk RNA-Seq Data Preprocessing and Analysis

For each sample, we performed gene expression quantification using RSEM (RNA-seq by Expectation Maximization) v1.3.1 with STAR (Spliced Transcripts Alignment to a Reference) v2.7.1a as the aligner [33,34]. Paired-end sequencing reads in raw FASTQ format were processed with the “rsem-calculate-expression” function. The raw FASTQ files were aligned to the GRCh38 genome reference, which was prebuilt using the “rsem-prepare-reference” function. Downstream analyses were performed on the resulting gene quantifications. We used the DESeq2 (1.30.1) method to identify differentially expressed genes (DEGs). Genes with fewer than ten raw counts across all samples were excluded from the analysis. To create the DESeq2 object, we used the “DESeqDataSetFromMatrix” function, specifying the design to indicate the various experimental conditions. DEGs were estimated using the “lfcShrink” function with a “normal” estimator [35]. We performed Gene Set Enrichment Analysis (GSEA) using the fgsea (v1.16.0) R package with 50 broad hallmark gene sets and thousands of Gene Ontology (GO) sets. For comparison, we selected DEGs (adj *p* values < 0.05) and ranked them by fold change. From the ranked list, we chose the top 100 genes for further over-representation analysis. GO term over-representation analysis was conducted using the “enrichGO” function from the clusterProfiler (v3.18.1) package using the org.Hs.eg.db (v3.12.0) annotation database [36]. In independent analyses, Qiagen Ingenuity Pathway Analysis (IPA) software (version 01-22-01) was used to identify condition-dependent changes in signaling networks.

### 2.4. Protein Expression Analysis

Cultured cells were paraformaldehyde-fixed. For tissue analysis, tumors were fixed in 10% neutral buffered formalin (NBF) for 72 hr for paraffin embedding and tumor cross-sections (5 μM) cut for histology. Trichrome staining was performed using a commercially available kit HT15-1KT (Sigma; St. Louis, MO, USA). Samples were analyzed by immunofluorescence (IF) as described [32,37,38,39]. Upon blocking, the following primary antibodies were used (4 °C, 12 h): E-cadherin (CDH1) antibody 610181 (BD Biosciences) at 1:100; Ki-67 antibody 14-5698-80 (Invitrogen; Carlsbad, CA, USA) at 1:500; SFRP4 antibody HPA009712 (Sigma) at 1:100; COL11A1 antibody orb767627 (Biorbyt; Wuhan, Hubei, China) at 1:100; and -GFP (Gene Tex, #GTX26673; Irvine, CA, USA) at 1:250. As secondary antibody, Donkey Alexa488-conjugated IgG A11055 (Invitrogen) at 1:200 or Cy3-conjugated IgG 711-166-152 (Jackson ImmunoResearch; West Grove, PA, USA) at 1:200 was used. IF images were acquired with Biotek Cytation5/Gen5™ Software (Version 3.08, Biotek; Winooski, VT, USA).

### 2.5. Cell Culture Functional Assays

Co-cultures were performed in triplicate by plating 25,000 GFP+ Capan-1 cells with/without 25,000 RFP+ ASCs in 24-well plates for 40 hr in FBS-free DMEM. After 40 hr in coculture, cells were trypsinized for transwell assays. For cell migration assay, 900 μL of DMEM/20% FBS was added to each well. Transwell inserts (8 μM, #353097, Corning; Corning, NY, USA) were then placed in each well. Trypsinized cells from cocultures were added to the transwells in 300 μL of FBS-free DMEM and cells were incubated for 5 hr. For cell invasion assays, transwell inserts were placed in 24-well plates and coated with 300 μL of chilled PBS containing 10 mg/mL of Matrigel (#354234, Corning). Plates were then incubated for 3 hr to solidify the Matrigel. Then, 900 μL of DMEM/20% FBS was added to each well. The Matrigel solution was aspirated from the transwells, trypsinized cells from coculture were added to transwells in 300 μL of FBS-free DMEM, and cells were incubated for 24 hr. For analyses, transwells were removed from the plates and the apical side of the membrane was cleaned using a cotton swab wetted with PBS. Cells that migrated to the basal side of the membrane were fixed in 70% ethanol for 15 min. Membranes were removed from the transwells using a razor blade and mounted basal side up on charged glass slides. GFP IF was performed, and then membranes were washed and counterstained with DAPI to visualize nuclei. 15 images (10×) were taken of each membrane using Cytation5 /Gen5™ software (Version 3.08) for quantification.

### 2.6. Mouse Models

All mouse experiments were approved by and performed in accordance with the University of Texas Health Science Center at Houston Institutional Animal Care and Use Committee. Mice were housed in a barrier facility with ad libitum access to food and water and were maintained on a 12 h light/dark cycle. Frozen sperm of SFRP4-ko mice [29] was provided by Jason Mastaitis (Regeneron; Tarrytown, NY, USA) and mice were re-derived by Charles River (Wilmington, MA, USA). Then, mice were back-crossed into the C57BL/6 background. KPC FC1242 cells, originally isolated from the KPC genetic murine model of PDAC [40], were used as described [38,39]. Mice were anesthetized using inhaled isoflurane and Ethiqa XR, then placed in lateral recumbency on their right side, shaved, and aseptically prepped. A small incision was made in the abdomen to retract the pancreas. Using a 28G needle and a 1 mL syringe 10^5^ KPC or KPC-*SMAD4*-ko cells were injected into the pancreas over 30 s. The abdominal musculature was sutured, and the skin was closed using metal wound clips. Upon necropsy, tumors were weighed. Livers were minced and digested with collagenase and dispase to quantify metastasis-derived cells by the colony formation assay as described [38] by plating suspensions (1 × 10^6^ cells/well) in triplicate in 6-well plates in DMEM supplemented with 20% FBS for 7 days. Upon fixation with cooled methanol (−20 °C) for 20 min, colonies were visualized using crystal violet staining.

### 2.7. Statistical Analysis

Microsoft Excel and Graphpad Prism (version 10.6.0) were used to graph data as mean ± SD and to calculate *p*-values using homoscedastic Student’s *t*-Test for comparison of two groups or One-Way ANOVA for comparison of >three groups. *p* < 0.05 was considered significant. The total sample size was at least *N* = 3 per group, and experiments were repeated at least twice with similar results.

## 3. Results

### 3.1. PDAC Cells Activate TGFβ, Wnt, and SHH Signaling; LINC01614, SFRP4, and ECM Genes in ASC

**Figure 1:**
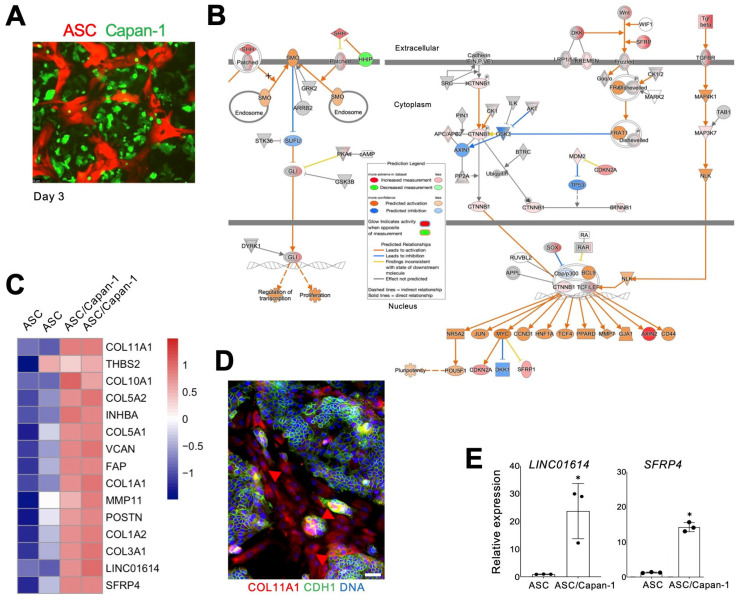
PDAC cell-induced ASC-CAF transition. (**A**), ASCs expressing RFP cultured with or without Capan-1 cells expressing GFP co-seeded at a 1:1 ratio. (**B**), Changes in RFP+ fibroblasts induced by Capan-1 co-culture after 7 days identified by IPA focused on SHH (right) and Wnt (left) signaling pathways. Changes in ASC/Capan-1 co-culture vs. ASCs alone (legend) indicate SHH and Wnt target activation. (**C**), The heatmap shows gene-wise scaled expression values of two potential therapeutic targets and aCAF genes [26] in two biological replicates of ASCs with/without Capan-1 cell co-culture. Scale bar: gene-wise z-score of relative gene expression from high (red) to low (blue). (**D**), IF analysis of ASC/Capan-1 co-culture demonstrating COL11A1 expression in CDH1-negative fibroblasts (red, arrowheads). CDH1+ (green): cancer cells. Blue: nuclei. Scale bar: 100 μM. (**E**), RT-PCR analysis of mRNA expression in RFP+ cells sorted from ASCs with/without Capan-1 co-culture demonstrating the induction of *LINC01614* and *SFRP4* expression. Plotted: mean +/− SD, * *p* < 0.05, Student’s *t*-test.

To investigate the effect of PDAC cells on ASCs, we used primary ASCs from human visceral AT. RFP-labeled ASCs were plated at semi-confluence alone or admixed 50:50 with Capan-1 cells expressing GFP. Cells have reached confluence by day 3 (Figure 1A). At day 7 of co-culture, ASC and Capan-1 cells were separated by FACS (Figure S1A) and processed for RNA sequencing. Ingenuity Pathway Analysis (IPA) identified “extracellular matrix organization” as the top significantly induced canonical pathway following co-culture with Capan-1 cells (*p* = 5 × 10^−21^). As top upstream regulatory pathways, beta-estradiol, TGFβ, TNF, and GLI were identified. Indeed, Capan-1 co-culture induced the expression of *TGFB3* by 7-fold (Table S1) and of *TGFB1* by 1.7-fold. Accordingly, the expression of TCF4 (3-fold) and other TCF/LEF transcription factors (TFs) downstream of Wnt and TGFβ signaling, was activated by Capan-1 co-culture. Gene expression analysis also confirmed a 7-fold induction of *GLI1*, the TF mediating Sonic Hedgehog (SHH) Hedgehog signaling. The induction of pathways engaging TGFβ, Wnt, and Hedgehog signaling in ASCs by Capan-1 cell co-culture is summarized in Figure 1B and Figure S1B.

Co-culture of ASCs with Capan-1 cells led to the upregulation of key genes associated with *COL11A1^+^THBS2^+^INHBA^+^* gene signature of aCAFs. Notably, 13 of the top 15 genes listed in Table 4 of Kim et al. [26] were strongly upregulated (Figure 1C). *COL11A1*, the most strongly induced gene, is activated at a late stage of the ASC-aCAF transition [23,27]. IF analysis confirmed COL11A1 expression in ASCs co-cultured with Capan-1 cells marked by CDH1 (Figure 1D). *COL11A1* and most other genes found induced by Capan-1 in ASCs (Figure 1C) have established roles in cancer progression. The exceptions are *LINC01614* and *SFRP4*, for which the function in PDAC CAFs has not been studied. Their upregulation in FACS-sorted ASCs following Capan-1 co-culture was validated both by RT-PCR in FACS-sorted ASCs (Figure 1E). Hence, we proceeded to interrogate their function.

### 3.2. LINC01614 or SFRP4 Ko in ASCs Prevents ECM Expression and Signaling Induced by PDAC Cells

**Figure 2:**
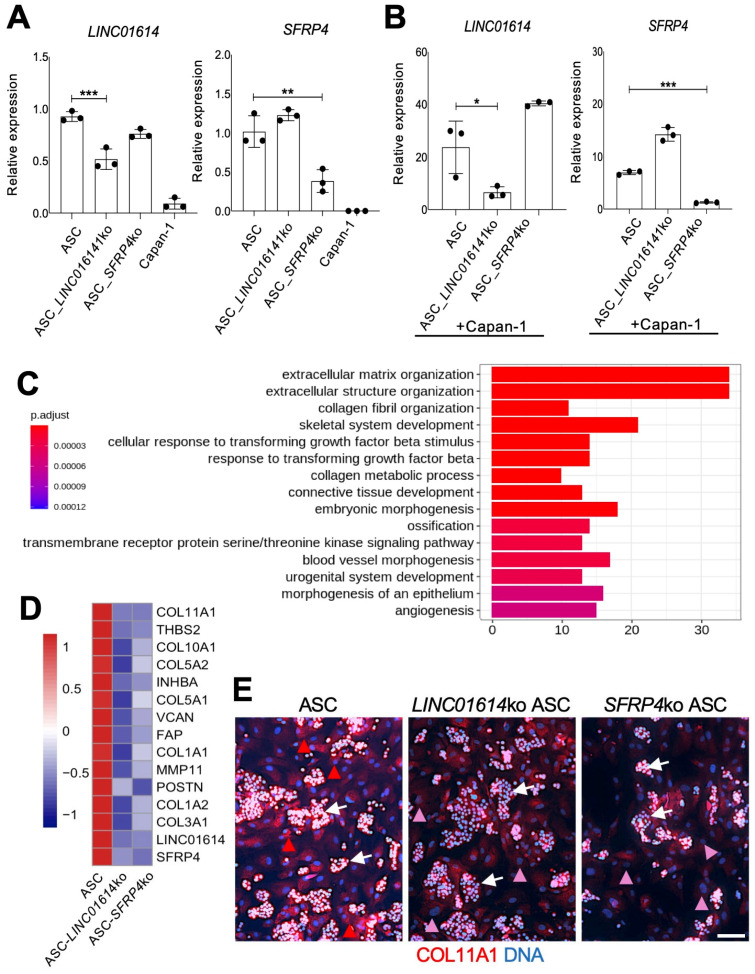
The effect of *LINC01614* ko and *SFRP4* ko in ASC-CAFs. (**A**), RT-PCR analysis of mRNA expression in parental, *LINC01614*-ko, and *SFRP4*-ko ASCs confirming reduction in the respective target gene expression. (**B**), RT-PCR analysis of mRNA expression in parental, *LINC01614*-ko, and *SFRP4*-ko RFP+ ASCs sorted from co-culture with GFP+ Capan-1 cells, confirming reduction in the respective target gene expression. A-B, Plotted: mean +/− SD, * *p* < 0.05, ** *p* < 0.01, *** *p* < 0.005, ANOVA. (**C**), Top 15 GO terms identified from the comparison between parental ASCs and *LINC01614*-ko ASCs after Capan-1 co-culture. DEGs were filtered based on adjusted *p*-values (<0.05) and ranked by log_2_ fold change, with the top 100 DEGs selected for analysis. (**D**), Knockout of *LINC01614* or *SFRP4* inhibits the expression of aCAF genes (Figure 1C) induced in Capan-1 co-culture, as measured by RNAseq data. The heatmap shows gene expression values averaged across samples within each condition. Expression values are scaled by gene-wise to highlight relative differences between conditions. Scale bar: gene-wise z-score of relative gene expression from high (red) to low (blue). (**E**), IF analysis of ASCs co-cultured with Capan-1 cells (arrows), revealing COL11A1 expression (red arrowheads) reduced (pink arrowheads) upon *LINC01614* ko and *SFRP4* ko. Blue: nuclei. Scale bar: 100 μM. White arrowheads: Capan-1 cell colonies.

Expression of *LINC01614* and *SFRP4* was undetectable by RT-PCR in Capan-1 cells (Figure 2A). To investigate the function of *LINC01614* and *SFRP4* in ASCs and CAFs, human visceral ASCs used in Figure 1 were transduced with CRISPR vectors to generate gene knockouts (kos). Proliferation of ASCs was not significantly affected by either *LINC01614* ko or *SFRP4* ko in Capan-1 co-culture (Figure S1A), which made it possible to isolate consistent numbers of cells for comparable analyses for each co-culture. RT-PCR confirmed that the baseline ASC expression of each gene was reduced by each respective ko (Figure 2A). The reduction in *LINC01614* and *SFRP4* expression in ASCs was significantly higher in co-culture with Capan-1 cells (Figure 2B).

We then subjected the *LINC01614*-ko and *SFRP4*-ko ASCs to RNA sequencing and comparative genomic analysis. To investigate the biological processes impacted by each ko, we performed GSEA. The hallmark pathways and GO enrichment analysis indicated a marked similarity between *LINC01614* ko and *SFRP4* ko effects (Figure S2A,B). Hallmark pathways, such as TGFβ signaling, Wnt signaling, EMT, angiogenesis, and hypoxia, were inhibited after either *LINC01614* or *SFRP4* ko (Figure S2A). There was also a concordance of pathways induced after both knockouts. The dependence of ECM remodeling on *LINC01614* and *SFRP4* was revealed from GO enrichment analysis (Figure 2C). Figure S2B summarizes it, demonstrating that each ko inhibited key processes related to ECM organization. Indeed, *COL11A1* and other collagen genes were not induced by Capan-1 co-culture upon either *LINC01614* or *SFRP4* ko in ASCs (Figure S2C). ECM organization, connective tissue activation, and fibrosis were also identified by IPA as the top processes dependent on both *LINC01614* (*p* = 7 × 10^−37^) and *SFRP4* (*p* = 4 × 10^−30^) in ASCs co-cultured with Capan-1, as shown in Figure S3A. Other IPA-identified pathways dependent on *LINC01614* and *SFRP4* related to cell migration, invasion, and metastasis (Figure S3B). GO over-representation analysis also identified the dependence of TGFβ signaling activation on target gene in Capan-1 co-culture (Figure 2C). That corresponded to the numbers of *TGFB1* and *TGFB3* reads being dramatically reduced by either *LINC01614* or *SFRP4* ko in RNAseq data. The dependence of TGFβ/Wnt signaling induction on *LINC01614* and *SFRP4* was also revealed by IPA focused on these pathways (Figure S3B). According to RNAseq data, *GLI1* expression was 9× lower in *SFRP4*-ko ASCs and 11× lower in *LINC01614*-ko ASCs than in parental ASCs co-cultured with Capan-1. This observation, also pinpointed by IPA in *SFRP4*-ko co-cultures (Figure S3A), suggests that the *LINC01614/SFRP4* pathway supports the Hedgehog-GLI1 signaling induced in ASCs by PDAC cells.

Top ASC genes found to be downregulated in *LINC01614*-ko in ASC co-cultured with Capan-1 cells were *HEPH*, *ID4*, *LINC01614*, *COL1A2*, and *FMOD* (Table S2). Top ASC genes found to be downregulated in *SFRP4*-ko in ASCs co-cultured with Capan-1 cells were *HEPH*, *OLFML3*, *ID4*, and *CHSY3* (Table S3). The overlap in downregulated gene lists suggested that ASC-expressed *LINC01614* and *SFRP4* operate within the same pathway. Notably, changes in *HEPH*, *ID4*, and other genes reduced in the *LINC01614*-ko and *SFRP4*-ko were not observed without Capan-1 co-culture in either *LINC01614* ko (Table S4) or *SFRP4* ko (Table S5) ASCs. Importantly, the expression on all aCAF signature genes (Figure 1C) was reduced upon either *SFRP4* or *LINC01614* ko in ASCs co-cultured with cancer cells (Figure 2D). The reduction in *COL11A1* expression upon *LINC01614* ko or *SFRP4* ko in Capan-1 co-culture was confirmed by IF (Figure 2E).

### 3.3. Co-Culture with ASCs Activates TGF/Wnt Signaling in PDAC Cells

**Figure 3:**
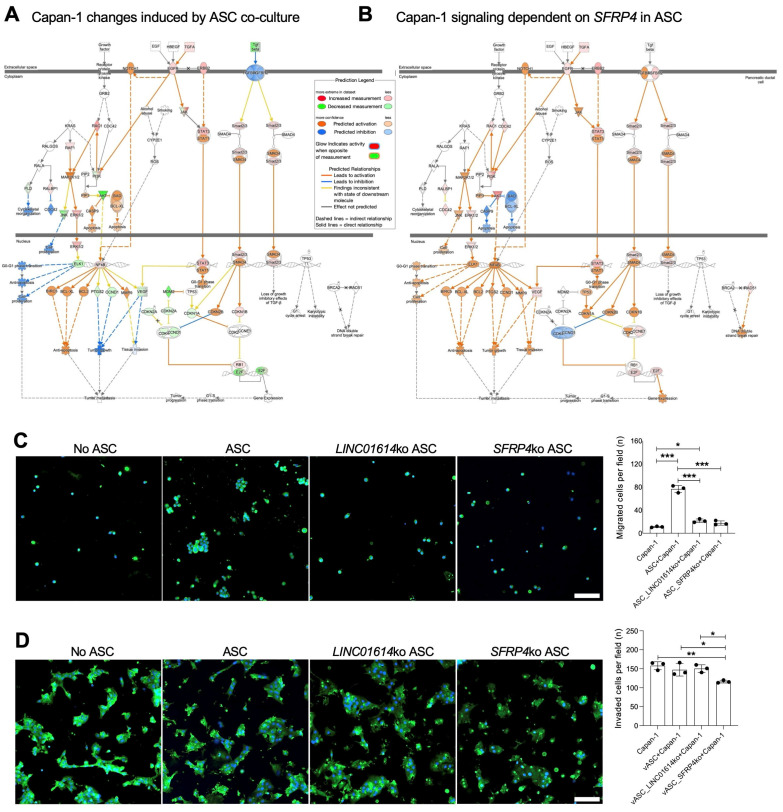
The effect of ASC-CAFs and their *SFRP4* and *LINC01614* expressed on PDAC cells. (**A**), Changes in GFP+ sorted Capan-1 cells induced by ASC-CAF co-culture after 7 days identified by IPA focused on pancreatic adenocarcinoma signaling pathways. Changes in co-cultured Capan-1 vs. Capan-1 alone (legend) indicate TGF target activation. (**B**), Changes in GFP+ Capan-1 cells in *SFRP4*-ko vs. parental ASC-CAF co-culture for 7 days identified by IPA focused on pancreatic adenocarcinoma signaling pathways indicate TGF target activation dependency on *SFRP4* expression. (**C**), Migration assay showing GFP+ cells in representative view fields of trans-well bottoms upon Capan-1 culture on the trans-well top with or without indicated RFP+ ASCs for 5 hrs. (**D**), Trans-well Matrigel invasion assay showing GFP+ cells in representative view fields of trans-well bottoms upon Capan-1 culture on the trans-well top with or without indicated RFP+ ASCs for 24 hrs. Blue: nuclei. Scale bar: 100 μM. Graphs: mean cell number/view field +/− SD, * *p* < 0.05, ** *p* < 0.01, *** *p* < 0.005, ANOVA.

Co-culture of with ASCs led to the activation of multiple signaling pathways in Capan-1 cells, indicating dynamic intercellular communication. Transcriptomics analysis identified Indian Hedgehog (*IHH*) among the top 10 and *SHH* among the top 20 genes induced by ASC co-culture (Table S6). Given that GLI1, the Hedgehog effector, is induced in ASCs by Capan-1 (Figure 1B), these data suggest IHH and SHH as PDAC-secreted factors that activate the ASC transition to CAFs. In addition to *IHH*, the top Capan-1 genes induced by ASCs in Capan-1 cells were *OLFM4*, *LGALS4*, *CA9*, *AC020978.6*, *LYZ*, and *NOXA1*. IPA identified “RHO GTPase cycle” as a top canonical pathway induced by ASCs (*p* = 2 × 10^−15^). As top upstream regulators, HNF4A, β-estradiol, K-RAS, and TP53 were identified by IPA. Activation of TGF-regulated networks was revealed in the analysis focused on “pancreatic adenocarcinoma signaling” pathways (Figure 3A). Activation of components of TGFβ/Wnt signaling, culminating in *HNF1A* induction, was also detected (Figure S3A).

### 3.4. LINC01614 or SFRP4 Ko in ASCs Prevents TGF/Wnt Signaling Induction in PDAC Cells

To investigate the function of ASC-expressed *LINC01614* and *SFRP4* in cancer progression, we analyzed Capan-1 cells co-cultured with the corresponding ko ASCs. *OLFM4*, *NOXA1*, and *IHH* were identified as the top three Capan-1 genes found to be dependent on ASC *LINC01614* from the dataset (Table S7). Remarkably, the same three genes, *OLFM4*, *NOXA1*, and *IHH*, were found to be dependent on ASC *SFRP4* (Table S8). This again demonstrates that *LINC01614* and *SFRP4* are parts of the same mechanism through which ASCs signal to PDAC cells. Notably, GLI expression is induced in ASCs co-cultured with Capan-1 cells (Figure 1B), whereas IHH is induced in Capan-1 cells co-cultured with ASCs. This suggests that IHH secreted by PDAC cells may be the signal through which the Hedgehog pathway is activated in CAFs. Activation of TGF-regulated networks, dependent on ASC-expressed *SFRP4*, was revealed in the analysis focused on “pancreatic adenocarcinoma signaling” pathways (Figure 3B). Activation of TGFβ/Wnt signaling, culminating in *HNF1A* induction, was also found to be dependent on ASC-expressed *LINC01614* and *SFRP4* (Figure S3B).

To determine the function of ASC-expressed *LINC01614* and *SFRP4*, we analyzed the effect of the knockouts on Capan-1 cells in co-culture. Proliferation of Capan-1 cells was not reduced by either *LINC01614* or *SFRP4* ko in co-cultured ASCs (Figure S1A). Co-culture with ASCs significantly increased the migration of Capan-1 cells through Matrigel in the trans-well assay (Figure 3C). Knockout of either *LINC01614* or *SFRP4* in ASCs reduced Capan-1 migration to baseline levels (Figure 3C). Interestingly, only SFRP4 knockout in ASCs significantly suppressed Capan-1 invasion (Figure 3D). These findings suggested that SFRP4 plays a broader role in regulating cancer cell aggressiveness.

### 3.5. SFRP4 Loss in the TME Suppresses PDAC Progression

**Figure 4:**
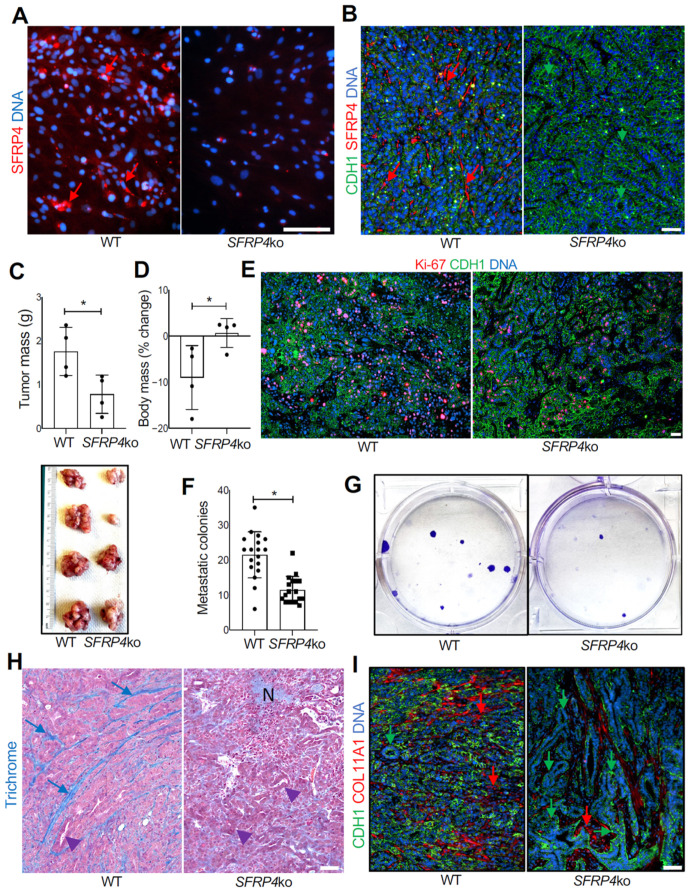
The effect of *SFRP4* ko on SMAD-dependent PDAC progression in mice. (**A**), IF on adherent ASCs demonstrating that SFRP4 expression (red arrows) is missing in cells from *SFRP4*-ko mice. (**B**), IF on KPC tumor sections demonstrating that SFRP4 expression (red arrows) is missing in CAFs of *SFRP4*-ko mice. CDH1+ (green): cancer cells. (**C**), Weights (mean +/− SD) and pictures of KPC tumors resected from WT and *SFRP4*-ko mice after 3 weeks. * *p* < 0.05, Student’s *t*-test. (**D**), Changes in body weight (mean +/− SD) from grafting day in WT and *SFRP4*-ko mice after 3 weeks. (**E**), IF on KPC tumor sections showing that the frequency of proliferating Ki-67+ cells (red) is lower in tumors of *SFRP4*-ko mice. (**F**), Quantification of metastatic KPC cells based on the numbers of colonies formed by adherent cells from liver cell suspensions. Graphs: mean colony number/well +/− SD. (**G**), Images of representative wells used for (**F**). * *p* < 0.05, Student’s *t*-test. (**H**), Representative sections of KPC tumors stained with Trichrome to reveal collagen deposition (blue arrows). N: necrosis; arrowheads: glandular epithelial structures increased in tumors of *SFRP4*-ko mice. (**I**), Representative sections of KPC tumors subjected to COL11A1 and CDH1 IF. Green arrows: epithelial CDH1 expression. Red arrows: stromal COL11A1 expression reduced in *SFRP4*-ko mice. Blue: nuclei. Scale bar: 100 μM.

We used a previously reported whole-body *SFRP4*-ko mouse strain [29] to test the role of host SFRP4 on PDAC progression. As a model of PDAC, we used cells from Kras^LSL-G12D^;p53^LoxP^;Pdx1-CreER (KPC) mice [40]. KPC cells were orthotopically grafted into the pancreas of *SFRP4*-ko and WT littermates, and cancer progression was monitored for 3 weeks. The loss of SFRP4 expression by ko ASCs was confirmed by IF on primary adipose-derived stromal/vascular cells adherent in culture (Figure 4A). The ko was also confirmed by IF on tumor sections, which also indicated that SFRP4 expression by cancer cells is negligible (Figure 4B). Tumor growth was significantly reduced by the host *SFRP4* ko (Figure 4C). Consistent with that, tumor-free body weight was significantly decreased due to cachexia in WT mice but not in *SFRP4* ko littermates (Figure 4D). Tissue section Ki-67 IF revealed decreased cancer cell proliferation in tumors of *SFRP4*-ko mice, accounting for slower tumor growth (Figure 4E). Although macro-metastases were not evident, we used a previously established colony formation assay [38] to assess micro-metastases. It revealed that metastatic cancer cell dissemination to the liver was significantly reduced by *SFRP4* ko in the TME (Figure 4F). Growth of cancer cell colonies from liver cell suspensions of *SFRP4*-ko mice was also lower in culture (Figure 4G). Desmoplasia, visualized by trichrome staining, was notably lower in tumors of *SFRP4*-ko littermates (Figure 4H). Specifically, stromal COL11A1 deposition was markedly lower in tumors of *SFRP4*-ko mice (Figure 4I). KPC tumors in *SFRP4*-ko littermates also had a higher presence of necrotic areas, indicating a decrease in cancer cell viability (Figure 4H). These differences were concomitant with a higher presence of glandular structures composed of cells with epithelial morphology, confirming a lack of dedifferentiation typical of aggressive PDAC (Figure 4H,I).

### 3.6. SMAD4 Loss in Cancer Cells Overrides the Effect of SFRP4 on Cancer Progression

**Figure 5:**
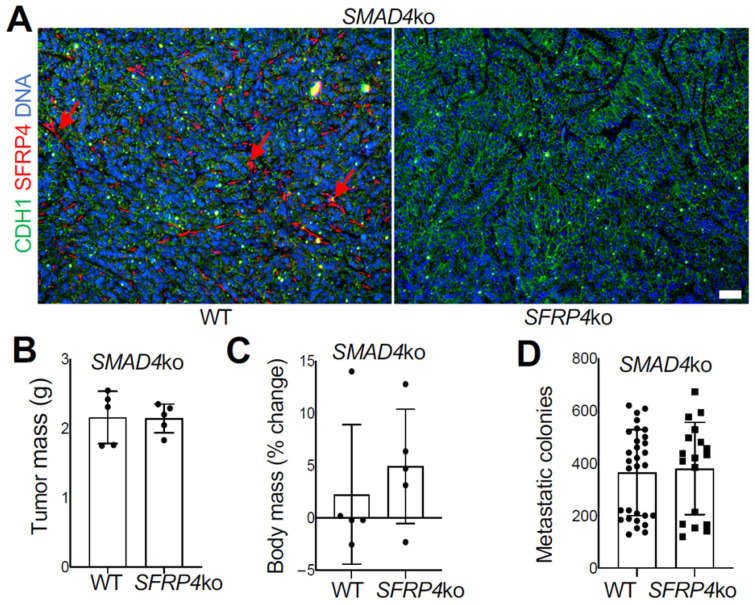

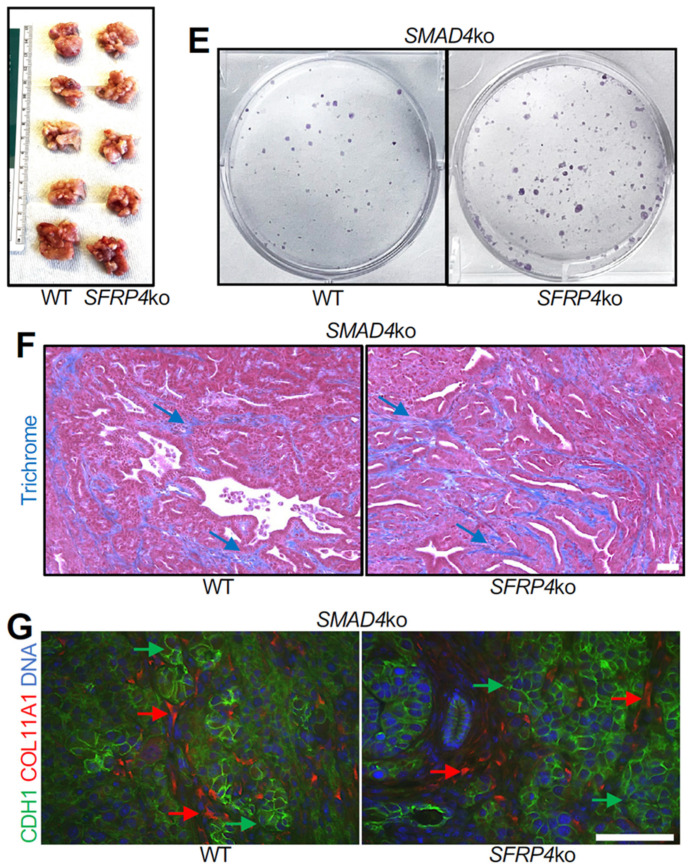
*SMAD4* ko overrides the SFRP4 dependency of PDAC progression in mice. (**A**), IF on *SMAD4*-ko KPC tumor sections demonstrating that SFRP4 expression (red arrows) is missing in CAFs of *SFRP4*-ko mice. CDH1+ (green): cancer cells. (**B**), Weights (mean +/− SD) and pictures of *SMAD4*-ko KPC tumors resected from WT and *SFRP4*-ko mice after 3 weeks. (**C**), Changes in body weight (mean +/− SD) from grafting day in WT and *SFRP4*-ko mice after 3 weeks. (**D**), Quantification of metastatic *SMAD4*-ko KPC cells based on numbers of colonies formed by adherent cells from liver cell suspensions. Graphs: mean colony number/well +/− SD. (**E**), Images of representative wells used for (**D**). (**F**), Representative sections of *SMAD4*-ko KPC tumors stained with Trichrome to reveal collagen deposition (blue arrows). (**G**), Representative sections of *SMAD4*-ko KPC tumors subjected to COL11A1 and CDH1 IF. Red arrows: stromal COL11A1 expression. Green arrows: epithelial CDH1 expression. Blue: nuclei. Scale bar: 100 μM.

Capan-1 cells used for human co-culture studies lack the expression of SMAD4, which is often observed in aggressive PDAC [41,42]. To simulate this hallmark in the murine model, we used *SMAD4*-ko KPC cells, which were orthotopically grafted into the pancreas of *SFRP4*-ko and WT littermates, and cancer progression was monitored for 3 weeks. As for parental KPC, SFRP4 ko was confirmed by IF on tumor sections (Figure 5A). In contrast to parental KPC, the growth of *SMAD4*-ko tumors was not reduced by *SFRP4* ko in the TME (Figure 5B). There was no difference in body weight loss between *SFRP4*-ko and WT littermates with *SMAD4*-ko tumors (Figure 5C). There was also no effect of *SFRP4* ko on liver metastases of *SMAD4*-ko tumors (Figure 5D). Growth of *SMAD4*-ko cancer cell colonies from liver cell suspensions of *SFRP4*-ko mice was also comparable in culture (Figure 5E). Desmoplasia, visualized by trichrome staining, was comparable in *SMAD4*-ko tumors of WT and *SFRP4*-ko littermates (Figure 5F). Specifically, there was no detectable difference in stromal COL11A1 deposition between WT and *SFRP4*-ko mice in *SMAD4*-ko tumors (Figure 5G). This indicates that the stimulatory effect of stromal SFRP4 on PDAC progression depends on functional SMAD4 in murine cancer cells.

## 4. Discussion

This study makes an important advance toward understanding the mechanisms through which ASC-derived CAFs regulate PDAC progression. The conversion of ASCs into CAFs, previously revealed by computational analysis of human genomic data [23], was simulated in human cell co-culture. While PDAC is highly heterogeneous, we used a controlled and reproducible co-culture system based on Capan-1 cells, which has limitations and advantages. We demonstrate for the first time that stromal *LINC01614* and *SFRP4* are required for the activation of TGF and Wnt signaling, as well as ECM production, by ASCs in the presence of cancer cells. *SFRP4* ko in mouse TME resulted in increased tumor cell death, reduced cancer cell proliferation and tumor growth, reduced desmoplasia, reduced cachexia, and a decrease in liver micro-metastases.

A previous study using subcutaneous ASCs immortalized by telomerase overexpression also concluded that Capan-1 cells could induce an ASC-aCAF transition [43]. However, the aCAF markers, such as *COL11A1*, *THBS2*, *MMP11*, and *SFRP4* [23,26] were not significantly induced in that study after co-culture [24]. Here, we have overcome the limitations of that study by using human ASCs derived from visceral adipose tissue, a depot physiologically relevant to PDAC. Importantly, we used primary ASCs without immortalization. The co-culture experiments resulted in the full transition to aCAFs with the computationally discovered aCAF pan-cancer gene signature described in our original study [26], which is marked by upregulation of *COL11A1*, *THBS2*, *INHBA*, *LINC01614*, *SFRP4*, *COL10A1*, and *MMP11* [23]. It remains debated how ASC-derived CAFs compare transcriptionally and functionally to other CAF populations in PDAC. As we recently reported [27], comparative analysis of gene expression profiles of ASC-derived CAFs with CAF transcriptomes reported by other studies will continue to bring clarity.

In our previous study, *LINC01614* was found associated with *COL11A1* across multiple cancer types (23). *LINC01614* has been reported as a promising diagnostic and prognostic marker in various cancers and linked to the tumor microenvironment and oncogenic function. Our previous study [23] identified *SFRP4* as a gene upregulated at the initiation of the transition from ASCs to aCAF, suggesting that it plays a role in triggering it. *SFRP4* has been indirectly implicated in various cancers, including pancreatic cancer, where its high expression was linked to EMT and poor patient prognosis [44]. The link between obesity/diabetes, the state of adipose remodeling, and PDAC aggressiveness is well established [8]. Overexpression of *SFRP4* in type-2 diabetes [45] is consistent with its potential role at an early stage if of ASC recruitment. However, the function of SFRP4 in pancreatic cancer has not been directly interrogated. Here, the observation that ASC-aCAF transition is inhibited in the absence of either *LINC01614* or *SFRP4* prompted us to investigate the mechanism of their function.

*LINC01614* has been previously reported to activate TGFβ signaling in PDAC [46]. Because TGFβ signaling is a well-established driver of fibrosis and cancer desmoplasia [47], it is likely that the induction of ECM genes is regulated by the *LINC01614/SFRP4* expression at least in part via TGFβ signaling back to CAFs (Figure S4). Our study identifies a number of other genes important to consider for a role in cancer-fibroblast crosstalk. The top fibroblast gene induced by Capan-1 co-culture is proteoglycan 4 (*PRG4*). While CAF-derived PRG4 has been shown to inhibit TGFβ and the progression of hepatocellular and breast carcinomas, a direct link of PRG4 to CAFs in PDAC is currently missing. The top genes found to be dependent on *LINC01614* and *SFRP4* in fibroblasts co-cultured with Capan-1 were *HEPH*, *ID4*, and *OLFML3.* HEPH codes for hephaestin, the plasma membrane ferroxidase that mediates the extracellular conversion of ferrous iron into its ferric form, protecting cells from ferroptosis [48]. Inhibitor of DNA binding 4 (ID4) promotes the proliferation, migration, and invasion of cancer cells [49]. *OLFML3*, coding for olfactomedin-3, has been implicated in cancer TGFβ and Wnt signaling. The exact roles of these CAF-expressed genes in PDAC are to be further investigated.

Our human genomic analysis reinforces the published evidence that *LINC01614* and *SFRP4* act through the Wnt pathway. Moreover, it suggests that *LINC01614* and *SFRP4* promote Wnt signaling in both CAFs and cancer cells. Wnt signaling is required for both initiation and progression of pancreatic cancer [50] in part by promoting the EMT [51]. It has been reported that *LINC01614* activates Wnt signaling by suppressing GSK3β [46]. SFRP proteins are generally thought to inhibit Wnt/β-catenin signaling by binding to Wnt ligands or Frizzled receptors [52]. However, some studies have suggested that SFRP4 may instead support Wnt signaling under certain conditions [53]. The Wnt-stimulating role of SFRP4 has been linked to its post-translational modifications and intracellular function [54]. In gastric cancer, increased SFRP4 expression was found to activate the Wnt pathway and promote tumor progression by antagonizing SFRP1 [55]. Specifically, there are correlative data suggesting that SFRP4 promotes Wnt signaling and metastases in PDAC [56]. While there is no known mouse ortholog of *LINC01614*, the mouse *SFRP4*-ko model enabled the interrogation of this pathway in vivo. Our mouse experiments demonstrate that it promotes cancer progression to liver metastases.

The model of multi-pronged effects of *LINC01614* and SFRP4 on cancer cells is proposed in Figure S5. While this model is supported by transcriptomic analyses, we note that the conclusions are primarily based on RNA-level data. Future work incorporating additional protein-level validation will be important to further clarify pathway directionality and causality. The synergistic effects of increased Wnt signaling, TGF signaling, and ECM remodeling are likely to be responsible for cancer progression, which is suppressed in the *SFRP4*-ko mouse model. The signaling changes induced by SFRP4 result in increased proliferation and invasiveness of PDAC cells, both facilitating metastases. Wnt and the TGF/SMAD pathways cross-talk in fibrogenesis [57] and their interdependence has been reported in pancreatic cancer [58]. Wnt signaling has been shown to mediate TGF/SMAD activation during myofibroblast proliferation [59]. Therefore, these pathways, regulated by LINC01614 and SFRP4, are likely to stimulate both CAFs and cancer cells. TGF/SMAD signaling is known to have stage-dependent and context-dependent effects in PDAC [60]. While initially it plays a tumor-suppressive role, in advanced cancer it has been shown to promote metastases [61]. Desmoplasia from CAF-secreted collagens is instrumental for metastatic dissemination [17,18]. COL11A1, induced by *LINC01614* and SFRP4, has been shown to induce EMT and invasiveness [62]. Emerging data from other cancers suggest COL11A1 as an important driver of disease aggressiveness induced by ASC-derived CAFs [63].

SMAD signaling is one of the key pathways dysregulated in PDAC [41]. Our data indicate that in mice SFRP4 metastasis-promoting function depends on SMAD4 expression in cancer cells. The mechanism through which *SMAD4* ko in cancer cells negates the dependency on SFRP4 is partly explained by previous studies. In the absence of SMAD4, non-canonical TGF signaling can become dominant and promote metastasis [64]. While TGF-induced EMT can drive cancer cells into apoptosis, in the absence of SMAD4, cell death is suppressed and EMT becomes unchecked and more aggressive, strongly contributing to the invasive and migratory phenotype of cancer cells [42]. Importantly, in human co-culture SFRP4 promotes invasiveness of Capan-1 cells, which are SMAD4-negative. This suggests that SFRP4 inhibition may suppress the aggressiveness of even SMAD4-negative human cancers. Tumor-targeted CRISPR-mediated inactivation of SFPR4 could be considered for future therapeutic approaches.

Downstream of the Wnt activation in cancer cells, our genomic analysis highlights the induction of *HNF1A*. This gene, coding for Hepatocyte Nuclear Factor 1-alpha, has been reported as both an oncogene and a promoter of cell proliferation and metabolism in PDAC [65]. Its potential importance in the process remains to be determined. The top genes expressed in Capan-1 found to depend on *LINC01614*/*SFRP4* in ASCs were *OLFM4*, *NOXA1*, and *IHH*, coding for Olfactomedin-4, NADPH oxidase activator 1, and Indian Hedgehog, respectively. OLFM4 has been uncovered as a potential prognostic PDAC biomarker and reported to promote cancer cell proliferation and chemoresistance [66]. NOXA1 regulates reactive oxygen species (ROS) and is hence likely important for cancer cell survival. Its expression in cancer correlates with increased tumor aggressiveness and poor prognosis, although it has not been specifically investigated in PDAC. It has been reported that IHH is induced in cancer cells and functionally contributes to PDAC progression via signaling to CAFs [67]. TGFβ signaling activates *IHH* transcription [68], which suggests a feedback loop in which SFRP4-driven TGFβ release from CAFs promotes cancer cell expression of IHH signaling back to CAFs (Figure S4). The Hedgehog effector GLI1, which we found to be induced by cancer cells, is known to promote fibroblast activation and ECM remodeling in PDAC [69]. Our data from human cell co-culture indicate that *LINC01614* and SFRP4 are important for both TGF signaling and GLI1 activation. Therefore, these genes appear to be the central perpetuators of this vicious cycle driving PDAC progression.

## 5. Conclusions

In summary, this study establishes a model of human ASC conversion into CAFs and the roles of *LINC01614* and *SFRP4* as key genes driving this process. It also discovers the function of these genes in activating Wnt and TGFβ signaling, desmoplasia, and metastatic cancer progression. The potential of SFRP4 as a drug target is to be further investigated. While conventional antiproliferative treatments are prone to resistance development due to selection pressure [70,71], blocking SFRP4 to suppress cancer cell invasiveness could offer an advantage.

## Data Availability

The datasets generated for this study can be found in the GEO database, accession GSE314456.

## Supplementary Materials

The following supporting information can be downloaded at: https://www.mdpi.com/article/10.3390/cancers18020233/s1. Table S1. ASC gene expression changes induced by Capan-1 co-culture. Comparison is for FACS-sorted RFP+ cells: ASC/Capan-1 vs. ASC alone. To remove the background of epithelial genes (transmitted by exosomes or/and from occasional Capan-1 cells stuck to RFP+ ACSs during FACS) 5438 genes differentially expressed by Capan-1 but not by ASCs (padj < 0.05; log2FoldChange > 0) were removed from the list. Shown are top genes most upregulated by Capan-1. Table S2. Expression changes upon *LINC01614* KO in ASCs co-cultured with Capan-1 cells. Comparison is for FACS-sorted RFP+ cells: ASC/Capan-1 vs. *LINC01614*-KO ASC/Capan-1. Shown are top genes most reduced by *LINC01614* KO. Table S3. Expression changes upon *SFRP4* KO in ASCs co-cultured with Capan-1 cells. Comparison is for FACS-sorted RFP+ cells: ASC/Capan-1 vs. *SFRP4*-KO ASC/Capan-1. Shown are top genes most reduced by *SFRP4* KO. Table S4. Expression changes upon *LINC01614* KO in ASCs cultured without Capan-1 cells. Comparison is for FACS-sorted RFP+ cells: ASC vs. *LINC01614*-KO ASC. Shown are top genes most reduced by *LINC01614* KO. Table S5. Expression changes upon *SFRP4* KO in ASCs cultured without Capan-1 cells. Comparison is for FACS-sorted RFP+ cells: ASC vs. *SFRP4*-KO ASC. Shown are top genes most reduced by *SFRP4* KO. Table S6. Capan-1 gene expression changes induced by ASC co-culture. Comparison is for FACS-sorted GFP+ cells: ASC/Capan-1 vs. Capan-1 alone. To remove the background of mesenchymal genes (transmitted by exosomes or/and from occasional ASCs stuck to GFP+ Capan-1 during FACS), 3764 genes differentially expressed by ASCs but not by Capan-1 cells (padj < 0.05; log2FoldChange > 0) were removed from the list. Shown are top genes most upregulated by ASCs. Table S7. Expression changes in Capan-1 cells upon *LINC01614* KO in co-cultured ASCs. Comparison is for GFP+FACS-sorted cells from co-cultures ASC/Capan-1 vs. *LINC01614*-KO ASC/Capan-1. To remove the background of mesenchymal genes (transmitted by exosomes or/and from occasional ASCs stuck to GFP+ Capan-1 during FACS), 3726 genes differentially expressed by ASCs but not by Capan-1 cells (padj < 0.05; log2FoldChange > 0) were removed from the list. Shown are top genes most reduced by *LINC01614* KO. Table S8. Expression changes in Capan-1 cells upon *SFRP4* KO in co-cultured ASCs. Comparison is for GFP+FACS-sorted cells from co-cultures ASC/Capan-1 vs. *SFRP4*-KO ASC/Capan-1. To remove the background of mesenchymal genes (transmitted by exosomes or/and from occasional ASCs stuck to GFP+ Capan-1 during FACS), 3967 genes differentially expressed by ASCs but not by Capan-1 cells (padj < 0.05; log2FoldChange > 0) were removed from the list. Shown are top genes most reduced by *SFRP4* KO. Figure S1. The effect of PDAC cell co-culture on ASCs. ASCs expressing RFP were cultured with or without Capan-1 cells expressing GFP, co-seeded at a 1:1 ratio. (A) After 7 days of co-culture, cells were FACS-separated and quantified (graphs). Plotted are mean values from three independent co-culture experiments and sorts. (B) Top pathway changes induced in ASC by Capan-1 co-culture identified in RNA-seq data by IPA. Activation of SHH signaling is highlighted. Figure S2. The effect of *SFRP4* and *LINC01614* ko on ASC gene expression in Capan-1 co-culture analyzed by GSEA. (A) Plot of NES and adjusted *p* values for the combined set of the top 10 most upregulated and top 10 most downregulated hallmark gene sets from each comparison indicated. The color scale represents the normalized enrichment score (NES) calculated by GSEA, while the circle size represents the significant level, measured by -log10 of the adjusted *p*-value. (B) Plot of NES and adjusted *p* values for the combined set of the top 10 most upregulated and top 10 most downregulated GO terms. (C) Expression of genes identified by Gene Set Enrichment Analysis (GSEA) for “GOMF extracellular matrix structural constituent conferring tensile strength” compared for parental, *LINC01614*-ko, and *SFRP4*-ko ASCs after co-culture with Capan-1. Heatmap shows gene-wise scaled average expression per condition. Scale bar: gene-wise z-score of relative gene expression from high (red) to low (blue). Figure S3. The effect of *SFRP4* and *LINC01614* ko on ASC gene expression in Capan-1 co-culture analyzed by IPA. (A) Top cellular processes and top canonical pathways downregulated by *LINC01614* ko and *SFRP4* ko in Capan-1 co-culture. TGFB1 and GLI1 signaling being centered on are highlighted. (B) Signaling focused on TGFβ and Wnt pathways, downregulated by *LINC01614* ko and *SFRP4* ko in Capan-1 co-culture. Figure S4. The effect of ASC, *SFRP4* ko in ASC, and *LINC01614* ko in ASCs on cancer cells. Data from total RNA expression comparison of Capan-1 co-cultured with parental ASCs vs. *LINC01614*-ko ASCs and vs. *SFRP4*-ko ASCs was analyzed by IPA to focus on the TGFβ/Wnt signaling pathways. (A) Capan-1 vs. Capan-1 + parental ASCs; (B) Capan-1 + parental ASCs vs. Capan-1 + *LINC01614*-ko ASCs; (C) Capan-1 + parental ASCs vs. Capan-1 + *SFRP4*-ko ASCs. Figure S5. The model of ASC-CAF cross-talk with PDAC cells. Upregulation of *LINC01614* and *SFRP4* by cancer cells induces Wnt signaling and the expression of TGFs and ECM in CAFs. TGFs and SFRP4 secreted from CAFs promote Wnt and TGF signaling in cancer cells, which synergize with desmoplastic changes, promote PDAC cell proliferation, survival, and migration, which collectively enable metastasis. Inactivation of SMAD4 short-circuits cancer cells to become more metastatic irrespective of SFRP4 signaling.
