# Supplementary material for: Adipose Stromal Cell-Derived Cancer-Associated Fibroblasts Promote Pancreatic Adenocarcinoma Progression Through SFRP4 Signaling
Source: Cancers (Basel). 2026 Jan 12;18(2):233. doi: 10.3390/cancers18020233 (PMC12838676; doi:10.3390/cancers18020233)
Supplement: Supplementary file 1 [file cancers-18-00233-s001.zip › cancers-4079074-supplementary.pdf]

cancers

MDPI

Supplementary Materials: Adipose Stromal Cell-Derived Cancer-Associated Fibroblasts Promote Pancreatic Adenocarcinoma Progression Through SFRP4 Signaling

Joseph Rupert, Lingyi Cai, Alexes Daquinag, Dimitris Anastassiou and Mikhail G. Kolonin

Table S1. ASC gene expression changes induced by Capan-1 co-culture. Comparison is for FACS-sorted RFP+ cells: ASC / Capan-1 vs ASC alone. To remove the background of epithelial genes (transmitted by exosomes or/and from occasional Capan-1 cells stuck to RFP+ ACSs during FACS) 5,438 genes differentially expressed by Capan-1 but not by ASCs (padj<0.05; log2FoldChange > 0) were removed from the list. Shown are top genes most upregulated by Capan-1.

	baseMean	log2FoldChar	lfcSE	stat	pvalue	padj
PRG4	228.44726	5.651362	0.7570271	5.0469276	4.49E-07	6.07E-06
COL11A1	677.82549	5.3646669	0.8828253	3.2753396	0.0010554	0.0058376
LINC01614	29.259781	5.0018249	0.5914399	5.6258466	1.846E-08	3.287E-07
NDNF	60.105648	4.9054574	0.7391953	5.4850846	4.133E-08	6.908E-07
COMP	644.32998	4.6820752	0.8098306	3.0580013	0.0022282	0.0108727
IL11	146.57479	4.4657191	0.6475872	4.4941373	6.985E-06	7.267E-05
COL10A1	45.298144	4.2714836	0.6458021	5.7560092	8.613E-09	1.623E-07
CDKN2B	814.18664	4.1493487	0.5369996	4.9966779	5.833E-07	7.732E-06
LINC01705	17.054966	4.101629	0.7762326	3.2568454	0.0011266	0.0061655
STC1	76.365306	4.0129448	0.7701069	3.1204059	0.001806	0.0090982
PRELP	565.1375	3.9939998	0.7252672	2.9259417	0.0034342	0.0155049
SYTL5	51.1383	3.9929215	0.5048256	5.913397	3.351E-09	6.879E-08
ISLR2	27.743585	3.9236637	0.5976447	5.5463983	2.916E-08	5.01E-07
CACNA1H	29.251909	3.8423728	0.6526312	4.6732549	2.965E-06	3.363E-05
MMP11	193.06265	3.6592067	0.5218609	5.0819994	3.735E-07	5.144E-06
SPON1	58.432498	3.6261646	0.4682933	5.8740644	4.252E-09	8.5E-08
SAMD11	100.22211	3.5845454	0.348873	8.0009892	1.234E-15	6.026E-14
ITGA1	850.07623	3.556242	0.2455746	12.995655	1.295E-38	3.482E-36
RP11-401P9.	26.077563	3.4395658	0.9004416	2.7626499	0.0057334	0.0235316
HS3ST3A1	75.779552	3.4202386	0.9089574	1.8203157	0.0687109	0.164694
MXRA5	1282.8224	3.4114065	0.611685	3.3849375	0.0007119	0.0041971
ADAMTS12	331.23119	3.3926325	0.3013896	9.4143488	4.76E-21	3.85E-19
ADAMTS4	77.452919	3.3883449	0.7028581	3.5196642	0.0004321	0.0027512
SFRP4	357.36755	3.386679	0.6457678	3.0958319	0.0019626	0.0097614
NKX3-2	35.802689	3.3712565	0.67888	2.4274059	0.0152072	0.0515614
DIO2	265.30085	3.3487114	0.7669579	3.7974102	0.0001462	0.0010645
RNF112	43.989948	3.3457481	0.6312554	3.6755531	0.0002373	0.0016285
FLJ16779	13.489062	3.3344029	0.7024731	3.8085506	0.0001398	0.0010235
ANOS1	30.083843	3.282678	0.5170668	5.6430486	1.671E-08	2.997E-07
TNFSF4	129.7109	3.2427373	0.6250705	3.2670418	0.0010868	0.0059871
MGP	2265.7069	3.1877091	0.4434362	4.826223	1.391E-06	1.688E-05
SSC5D	358.8656	3.1589824	0.5595981	3.3159427	0.0009133	0.0051705
MMP16	145.76777	3.141748	0.5591694	3.4297965	0.000604	0.0036442
IL16	43.254065	3.1289151	0.6689828	3.1815641	0.0014648	0.0076629
HOPX	37.66413	3.0690271	0.6383072	3.7501683	0.0001767	0.0012573
ISLR	1672.2865	3.0640851	0.3650855	5.8162243	6.019E-09	1.168E-07
WISP1	19.359582	3.0422243	0.7629184	1.9415371	0.0521932	0.134383
CST2	36.533311	3.0220427	0.6433251	2.7299222	0.0063349	0.0254906
INHBE	26.666263	3.0140251	0.7010178	2.2813246	0.0225292	0.0705828
CARMN	470.36411	2.9558315	0.5449501	3.8208393	0.000133	0.0009795
PRR5L	49.085248	2.9442241	0.4019587	5.5207155	3.376E-08	5.724E-07
C1QTNF5	33.486979	2.938343	0.6123962	2.7451084	0.0060491	0.0245683
IGF1	29.300335	2.9270897	0.9178948	1.5780313	0.1145584	0.2413693
FMOD	2322.9642	2.9092213	0.4263306	4.312593	1.614E-05	0.0001538
SCG2	195.77988	2.8719126	0.6154692	3.0093387	0.0026182	0.0124108
CASC15	22.782099	2.8537868	0.7044891	2.8848049	0.0039166	0.0171851
ADAMTSL2	18.146603	2.852479	0.845174	1.5068678	0.1318445	0.2677711
FNDC1	91.697463	2.8406663	0.9065071	1.315917	0.1882019	0.3459732
BAALC	19.745355	2.8390374	0.7902546	2.0974675	0.0359522	0.1010293
SRPX2	194.24431	2.8178677	0.300848	7.750407	9.16E-15	4.066E-13
TGFB3	119.45936	2.7796329	0.431487	5.2653679	1.399E-07	2.103E-06
GLI1	48.121487	2.773586	0.523499	3.0748281	0.0021062	0.0103624

 https://doi.org/10.3390/cancers18020233

Table S2. Expression changes upon *LINC01614* KO in ASCs co-cultured with Capan-1 cells. Comparison is for FACS-sorted RFP+ cells: ASC / Capan-1 vs *LINC01614*-KO ASC / Capan-1. Shown are top genes most reduced by *LINC01614* KO.

	baseMean	log2FoldChan	lfcSE	stat	pvalue	padj
HEPH	285.194603	5.16171671	0.29491768	8.1517513	3.5869E-16	7.0754E-14
ID4	186.656297	5.10377411	0.29467488	5.11304257	3.1701E-07	9.6533E-06
LINC01614	29.2597809	4.92677802	0.45873608	4.15933785	3.1917E-05	0.00050233
COL1A2	77470.1939	4.84229356	0.31321685	6.72234414	1.7882E-11	1.4975E-09
FMOD	2322.96422	4.81974421	0.35847618	6.107853	1.0098E-09	5.6911E-08
COL3A1	31940.5482	4.77052097	0.33710468	5.91716615	3.2754E-09	1.6614E-07
EMILIN1	1077.33159	4.74949815	0.18891378	17.6195637	1.7434E-69	3.0951E-65
CD248	1795.73316	4.7449696	0.4618012	6.19767055	5.7305E-10	3.3798E-08
SAMD11	100.222106	4.69571631	0.303194	5.55363532	2.7979E-08	1.1289E-06
NPR3	2543.49891	4.69032556	0.43816001	5.34867177	8.8602E-08	3.1334E-06
SMAD9	186.744399	4.6894074	0.28322512	8.16338938	3.2575E-16	6.4978E-14
PRG4	228.447261	4.67969921	0.52346684	4.25536685	2.0871E-05	0.00034823
PRELP	565.137501	4.65778227	0.51580096	4.47879469	7.5066E-06	0.00014824
SULF1	8954.32674	4.62236763	0.3141428	6.60247099	4.0436E-11	3.1085E-09
VCAN	21089.5698	4.61555075	0.2590311	8.74781398	2.1753E-18	6.775E-16
PTK7	1215.68308	4.60528072	0.29446893	9.15040655	5.672E-20	2.1424E-17
COL5A1	15036.3778	4.60232509	0.3728828	5.15553951	2.529E-07	7.9465E-06
EDNRA	57.747651	4.5159882	0.42305242	4.45551542	8.3692E-06	0.00016215
CHSY3	91.2928788	4.45873551	0.34427212	5.50746647	3.6403E-08	1.433E-06
COL1A1	145670.56	4.44332969	0.30352034	6.94364076	3.8212E-12	3.6277E-10
TSHZ3	156.161536	4.41881655	0.26555121	8.13426157	4.1446E-16	8.0856E-14
SV2A	84.7588455	4.39940018	0.32043454	5.92554283	3.1127E-09	1.5971E-07
COL6A3	29368.9943	4.37791309	0.36216645	4.49661076	6.9045E-06	0.00013795
ISLR	1672.28653	4.32914387	0.31399448	7.13537189	9.6526E-13	1.0449E-10
COL5A2	7681.19243	4.27636339	0.329055	5.75318261	8.7579E-09	3.9764E-07
HIC1	237.204929	4.2730225	0.2354233	9.94557063	2.6366E-23	1.8003E-20
MFAP4	861.195202	4.24710521	0.31537535	6.40694003	1.4847E-10	9.7621E-09
COL15A1	2879.62555	4.24313148	0.42678251	4.68597376	2.7863E-06	6.2694E-05
KLHL9	176.488953	4.23974802	0.2418254	8.31389482	9.2611E-17	2.0298E-14
THBS2	7491.43899	4.23850932	0.38912471	4.74366283	2.0989E-06	4.9353E-05
C1QTNF5	33.4869793	4.23027101	0.47373265	3.96513779	7.3354E-05	0.00100327
OLFML3	199.492823	4.21827865	0.28293317	6.28676638	3.2415E-10	1.9981E-08
CDKN2B	814.186636	4.2106619	0.43037852	3.3141665	0.00091917	0.00772076
LAMA2	521.971912	4.20049985	0.32687234	7.67978802	1.5935E-14	2.3802E-12
CPZ	180.638193	4.18421109	0.27153173	8.42677959	3.5531E-17	8.524E-15
FBLN1	696.706767	4.18253174	0.34251907	5.32848617	9.9035E-08	3.4746E-06
COL4A1	13651.4702	4.18142772	0.38531104	4.30696241	1.6551E-05	0.00028807
TBX3	62.9325084	4.17030382	0.33552758	4.77676919	1.7813E-06	4.3026E-05
PCDHGA10	152.851581	4.16369829	0.33566622	6.49239673	8.4481E-11	5.9992E-09
AEBP1	590.207713	4.12851411	0.47874154	4.71169851	2.4566E-06	5.6274E-05
TCF21	252.396797	4.12439291	0.25502115	8.23648928	1.7734E-16	3.6608E-14
PKDCC	247.69219	4.12247197	0.27105435	7.5825381	3.3886E-14	4.8514E-12
MMP16	145.767765	4.11773238	0.44452091	5.37300098	7.7437E-08	2.7885E-06
COL4A2	11957.3509	4.10568395	0.33883343	4.98156141	6.3073E-07	1.7414E-05
SPON1	58.4324982	4.0860961	0.38857518	4.93137403	8.1653E-07	2.1798E-05
INHBA	4268.82938	4.0654011	0.32180147	5.33290562	9.6654E-08	3.3978E-06
FN1	295979.817	4.0488303	0.31733713	5.60208832	2.1178E-08	8.7846E-07
LUM	2188.3394	4.01511196	0.21859671	11.377112	5.4371E-30	7.4249E-27
XIST	1347.14708	3.99848388	0.37526657	4.20142546	2.6524E-05	0.00042868
CDH11	3190.54776	3.99791533	0.3806397	4.26515101	1.9977E-05	0.00033616

https://doi.org/10.3390/cancers18020233

Table S3. Expression changes upon SFRP4 KO in ASCs co-cultured with Capan-1 cells. Comparison is for FACSsorted RFP+ cells: ASC / Capan-1 vs SFRP4-KO ASC / Capan-1. Shown are top genes most reduced by SFRP4 KO.

	baseMean	log2FoldChan	lfcSE	stat	pvalue	padj
HEPH	285.194603	4.84821152	0.28336273	11.1766772	5.3039E-29	4.0498E-26
OLFML3	199.492823	4.58872982	0.26847683	7.47875751	7.5029E-14	6.8201E-12
ID4	186.656297	4.33562604	0.2855348	9.17809293	4.3879E-20	1.2139E-17
LINC01614	29.2597809	4.22641506	0.40288009	5.19745483	2.0204E-07	4.845E-06
CHSY3	91.2928788	4.20751119	0.32500416	8.22729671	1.9149E-16	2.9961E-14
SAMD11	100.222106	4.1325634	0.29460795	8.86852914	7.4118E-19	1.7204E-16
PKDCC	247.69219	4.04419176	0.26220335	10.9781383	4.8685E-28	2.9979E-25
TSHZ3	156.161536	4.00306443	0.25734628	10.2162517	1.677E-24	7.1139E-22
TBX3	62.9325084	3.97105505	0.317844	7.46056847	8.615E-14	7.7207E-12
NPR3	2543.49891	3.94871891	0.39405197	5.17081868	2.3307E-07	5.5131E-06
SV2A	84.7588455	3.93172059	0.30596667	7.47650418	7.6326E-14	6.9051E-12
SMAD9	186.744399	3.9213065	0.2746248	8.95714787	3.3318E-19	8.1541E-17
CD248	1795.73316	3.88373856	0.40710424	4.83916978	1.3038E-06	2.4716E-05
PRG4	228.447261	3.88312236	0.42644883	5.15921778	2.4798E-07	5.8154E-06
PCDHGA10	152.851581	3.86860125	0.3195809	8.49971257	1.9006E-17	3.6647E-15
EDNRA	57.747651	3.82846728	0.38293742	5.29382251	1.1979E-07	3.011E-06
FBLN1	696.706767	3.81778244	0.32615535	7.46417711	8.3822E-14	7.5475E-12
FMOD	2322.96422	3.79132509	0.33913972	5.34058117	9.2649E-08	2.4128E-06
PRELP	565.137501	3.76127018	0.42700621	5.33711655	9.4436E-08	2.4526E-06
SPON1	58.4324982	3.70590891	0.35962848	6.86407007	6.6926E-12	4.4359E-10
C11orf96	35.3158004	3.62960636	0.35822842	5.74646147	9.113E-09	3.0839E-07
PTK7	1215.68308	3.61750859	0.28521447	7.58218222	3.3979E-14	3.3093E-12
LAMA2	521.971912	3.58164648	0.31392408	7.23006435	4.8277E-13	3.8398E-11
COL3A1	31940.5482	3.57022495	0.32229803	4.86392934	1.1508E-06	2.2122E-05
CDKN2B	814.186636	3.56165305	0.38934216	3.93588734	8.289E-05	0.00087369
POSTN	3149.98042	3.56135211	0.33391817	5.61228532	1.9967E-08	6.228E-07
HIC1	237.204929	3.55951305	0.227827	11.1072784	1.1563E-28	8.4895E-26
C1QTNF5	33.4869793	3.55036708	0.41148325	4.30196916	1.6929E-05	0.00022394
COL1A2	77470.1939	3.50991297	0.30197549	5.24801371	1.5375E-07	3.787E-06
KLHL9	176.488953	3.50008251	0.23436295	9.79179853	1.221E-22	4.3498E-20
SULF1	8954.32674	3.45482815	0.30247674	5.49926024	3.8139E-08	1.0981E-06
VCAN	21089.5698	3.44030914	0.24920152	7.8167239	5.4216E-15	6.5302E-13
TWIST1	57.359294	3.41351185	0.34520018	5.68733253	1.2904E-08	4.2106E-07
AEBP1	590.207713	3.41110608	0.41541359	4.07333421	4.6345E-05	0.00052754
COL6A3	29368.9943	3.40972915	0.3422107	4.17052307	3.039E-05	0.00036983
KIF26B	328.62837	3.39484444	0.36699357	6.18603305	6.1697E-10	2.7389E-08
NOTUM	29.1242874	3.37870774	0.39932101	5.52212797	3.3492E-08	9.8207E-07
IL11	146.574794	3.3763702	0.41901037	4.50666943	6.5853E-06	0.00010073
SLC2A3	384.057872	3.37503178	0.36531746	5.34776785	8.9046E-08	2.3221E-06
MFAP4	861.195202	3.37246776	0.30344703	5.9723949	2.338E-09	8.9617E-08
MMP16	145.767765	3.35225099	0.39745679	3.86812806	0.00010967	0.00110246
COL5A1	15036.3778	3.34138443	0.35021822	3.66864845	0.00024384	0.00217301
ADAMTS12	331.23119	3.32222283	0.26012038	8.98913602	2.4918E-19	6.2587E-17
MMP11	193.062647	3.31188709	0.38294198	5.46082097	4.7394E-08	1.3362E-06
INHBA	4268.82938	3.29988968	0.30947528	5.43428428	5.5017E-08	1.5203E-06
COL5A2	7681.19243	3.29337204	0.31568475	4.96772928	6.7741E-07	1.4056E-05
ISLR	1672.28653	3.27940906	0.30274042	5.56860726	2.5678E-08	7.8178E-07
ZNF626	24.3986536	3.26323607	0.40096463	4.74049084	2.132E-06	3.7823E-05
CPZ	180.638193	3.26132466	0.26459095	7.54710659	4.4503E-14	4.2265E-12
IL21R	45.854517	3.25662332	0.31937654	5.10884675	3.2413E-07	7.3484E-06

Table S4. Expression changes upon *LINC01614* KO in ASCs cultured without Capan-1 cells. Comparison is for FACS-sorted RFP+ cells: ASC vs *LINC01614*-KO ASC. Shown are top genes most reduced by *LINC01614* KO.

	baseMean	log2FoldChan	lfcSE	stat	pvalue	padj
SRGN	95.9643249	1.63107516	0.30976888	3.44310771	0.00057507	0.02015032
HAS2	232.338971	1.50593745	0.32356526	2.37308318	0.01764029	0.1571322
CXCL12	113.787016	1.43199518	0.30923608	1.74523117	0.08094464	0.3724316
ANGPT1	75.9006353	1.35639868	0.32339276	2.32187974	0.02023941	0.16893569
FAM180A	89.8198944	1.34391524	0.31921031	1.4433107	0.14893297	0.50777852
TBC1D2	608.414371	1.32642766	0.30874178	3.35952147	0.00078078	0.02444657
RN7SL471P	84.5069047	1.32076394	0.32309409	2.20296177	0.02759744	0.20194174
CD248	1795.73316	1.28401041	0.31863241	0.63695262	0.52415571	0.85747655
TFPI2	791.347928	1.26400895	0.31651686	1.14944696	0.25037173	0.64729053
PTGFR	38.0667412	1.24952834	0.32382661	1.13591413	0.2559925	0.65149861
TBXA2R	30.0094303	1.24583947	0.32139708	2.10340531	0.03543035	0.23241372
PCOLCE2	231.477477	1.24529531	0.30613868	2.66440201	0.00771253	0.09705511
ABI3BP	1269.29624	1.23801083	0.32357027	0.97500228	0.32955913	0.7252514
TEK	90.9452142	1.22665422	0.31463633	2.05896387	0.0394977	0.24770119
SCUBE3	1814.87422	1.20261343	0.3191354	1.92716382	0.05395922	0.29565018
CADPS	72.3546598	1.18759438	0.29657108	1.32003454	0.18682349	0.56481586
MIR137HG	65.3690141	1.17623021	0.32353981	1.71431289	0.08647128	0.38590762
EBF3	27.0068366	1.16110957	0.31999054	1.20196613	0.22937665	0.62369867
KIF20A	323.82275	1.14682772	0.27382898	4.52228372	6.1176E-06	0.00179522
HHIP	1165.14639	1.13949716	0.32073727	1.14249154	0.25324976	0.64898046
MYPN	53.3086572	1.13551504	0.31345578	1.58040887	0.11401326	0.44490618
RGS4	2084.80867	1.11625669	0.31015553	0.71601919	0.4739795	0.83073082
HGF	96.7365608	1.11403716	0.26669841	1.18612666	0.23557228	0.631335
RELN	521.072304	1.09607794	0.31131911	0.1257571	0.8999242	0.97825598
B3GALT2	48.2006039	1.08377021	0.32230981	1.31283771	0.18923764	0.56880695
ITGA10	40.6345285	1.08261243	0.32182863	1.45025832	0.1469865	0.5049172
COL7A1	876.353661	1.07834201	0.2612469	4.13533359	3.5444E-05	0.00411009
PDE3A	77.4579336	1.06379252	0.32326951	1.07951617	0.28035769	0.67623329
ARHGAP22	194.48675	1.05583094	0.30101254	2.25971968	0.02383865	0.18576372
SLC14A1	23.819165	1.0452538	0.27042838	1.20259602	0.22913269	0.6236106
COLEC10	44.2092325	1.04241674	0.32327856	0.28435405	0.77613907	0.95796711
GDF6	168.738017	1.04185083	0.32147752	0.37810212	0.70535474	0.93515592
RTN1	188.751609	1.03277694	0.32106414	0.74742357	0.45480792	0.81761966
FGF5	357.279743	1.0298543	0.27090402	1.65563422	0.09779592	0.41182444
CDH18	22.9360814	1.02091735	0.31773138	0.27488873	0.78340172	0.95973633
MIR31HG	11.6616389	1.01449253	0.30640497	1.08216162	0.27918072	0.67438238
SLIT2	244.712842	1.01159588	0.31187226	1.04855647	0.2943823	0.69016239
COL22A1	104.448768	1.00140612	0.28396576	1.24394955	0.21351813	0.60250147
PLCXD3	70.5797632	1.00017791	0.31746378	3.48979766	0.00048339	0.01819098
DNASE1L2	6.1134267	0.99805226	0.27108842	2.8212151	0.00478421	0.0731026
CST1	522.413374	0.99525795	0.32351149	2.04544586	0.04081092	0.25267984
SFRP1	540.102547	0.99443652	0.32157171	0.95331733	0.34042931	0.73548509
STEAP1B	52.2521019	0.99112825	0.32218385	0.61805836	0.53653687	0.86352675
AEBP1	590.207713	0.98991237	0.31373646	0.66057645	0.50888397	0.84822582
ABHD14A-ACY	19.1679384	0.98426071	0.27758603	3.46327604	0.00053364	0.01930978
CCNB1	611.552269	0.97965476	0.23106257	4.37647916	1.2061E-05	0.00253503
HIST1H1A	22.9485309	0.97782945	0.3030631	0.49159521	0.62300553	0.90458737
CBS	257.081952	0.97646334	0.32319612	2.35853957	0.018347	0.16096435
PDCD1LG2	41.1020598	0.96672712	0.32342671	0.7180022	0.47275593	0.82980641
ADAMTSL1	499.89241	0.96537343	0.30120275	0.96825058	0.33291924	0.72831315

Table S5. Expression changes upon *SFRP4* KO in ASCs cultured without Capan-1 cells. Comparison is for FACS-sorted RFP+ cells: ASC vs *SFRP4*-KO ASC. Shown are top genes most reduced by *SFRP4* KO.

	baseMean	log2FoldChan	lfcSE	stat	pvalue	padj
COLEC10	44.2092325	1.46168987	0.30184946	2.39386405	0.01667193	0.25225458
CTSK	139.601246	1.43428057	0.28780353	3.99381624	6.5018E-05	0.01442836
DSG2	872.778859	1.30030738	0.16587982	8.24723894	1.621E-16	1.4388E-12
RELN	521.072304	1.29442972	0.28643788	1.06146072	0.28848057	0.80089012
CEACAM6	5356.60781	1.28083213	0.25526859	5.71537325	1.0946E-08	2.1592E-05
HIST1H2AJ	583.985853	1.24980907	0.19324736	6.96978565	3.1742E-12	1.8784E-08
CDKN2D	68.6177398	1.22727247	0.28100703	4.576149	4.7361E-06	0.00280269
CCDC3	297.678959	1.18832067	0.30058792	1.38384134	0.16640704	0.66902459
NPTX1	180.647655	1.17739631	0.29666943	6.2671737	3.6766E-10	1.0878E-06
SHE	32.1972967	1.1530407	0.29108606	1.47472733	0.14028587	0.63073586
RASSF2	202.962968	1.13038805	0.22778193	4.68518393	2.7971E-06	0.00180498
HIST1H2BO	307.615512	1.12793836	0.23434826	5.59478984	2.2089E-08	3.3382E-05
CADPS	72.3546598	1.12511686	0.27065524	1.59015433	0.11180002	0.57674486
ALDH1A1	663.89737	1.1165262	0.24762602	5.59109354	2.2564E-08	3.3382E-05
PCSK9	751.155634	1.11339723	0.19192877	6.03188354	1.6206E-09	3.961E-06
PTGFR	38.0667412	1.11080912	0.30421229	0.96928804	0.33240149	0.83503151
ITGA10	40.6345285	1.08965836	0.30051301	1.85247171	0.06395812	0.46062817
FAM180A	89.8198944	1.08898517	0.3042249	0.83155403	0.40566072	0.88016694
PID1	273.344523	1.07620418	0.30351779	1.01080749	0.31210858	0.82017184
TFPI2	791.347928	1.07036221	0.29256858	0.76197054	0.44607759	0.90547435
CHAC1	106.233288	1.05606828	0.3035488	3.9944772	6.4837E-05	0.01442836
ABCC9	127.795129	1.04615207	0.26159467	2.75377342	0.00589125	0.15304971
FGF13	18.4800008	1.04388313	0.27380964	2.91589846	0.00354666	0.11813103
GDF5	55.3489775	1.03418324	0.29635396	0.3763206	0.70667857	0.99756199
CCNA2	275.755015	1.03291066	0.22758592	4.73395163	2.2019E-06	0.00150348
GREM1	8914.92826	1.02606326	0.28787504	0.2506039	0.80212036	0.99999694
TCEAL7	47.6484486	1.015213	0.30345323	1.45413028	0.14591019	0.63926376
FAM43A	322.455257	1.0083216	0.3017218	2.73364598	0.00626374	0.15728443
SLC14A1	23.819165	1.00062342	0.2445558	1.73922649	0.08199493	0.51132757
SRGN	95.9643249	0.99161693	0.28508825	0.29864097	0.765214	0.99999694
FENDRR	73.4112325	0.98805958	0.29896574	1.56550043	0.11746562	0.58794615
STC2	4548.46423	0.98325944	0.27874494	0.29452931	0.76835347	0.99999694
RPL41P2	16.4105299	0.98244853	0.30254263	3.12865089	0.00175611	0.08300887
CLDN11	95.0499981	0.98003569	0.30313314	2.70700544	0.00678931	0.1643011
HAS2	232.338971	0.97631366	0.30398965	0.52115614	0.602258	0.96955574
FGF7	211.731835	0.97493091	0.30152452	0.87780053	0.38005198	0.86345924
TMEM126A	71.2371531	0.97324205	0.25651964	3.44821504	0.0005643	0.04659581
HSD11B1	50.701984	0.96822097	0.30395048	2.13139295	0.03305678	0.34911188
HIST1H1A	22.9485309	0.9646974	0.27760163	0.8025939	0.4222095	0.88977744
CCNB1	611.552269	0.95684841	0.22650848	4.35093276	1.3556E-05	0.00523172
PTGS1	909.0954	0.95634763	0.28808139	1.29748981	0.19446271	0.70525473
FGF5	357.279743	0.95531151	0.26438918	1.55068343	0.12097757	0.59493484
CDH18	22.9360814	0.95251962	0.29446107	0.35834803	0.72008288	0.99999694
CXCL12	113.787016	0.95095107	0.29727257	0.35205382	0.7247979	0.99999694
SFRP1	540.102547	0.94745708	0.29928327	1.04829129	0.29450442	0.80473095
RTN1	188.751609	0.94493329	0.29847614	0.71810451	0.47269285	0.92018538
SESN2	201.802448	0.94079277	0.26992044	3.10248588	0.00191903	0.08646824
ELN	372.251004	0.93838936	0.26792734	0.39702598	0.6913483	0.99303283
PLCXD3	70.5797632	0.93532773	0.30309541	3.25662163	0.00112747	0.06849785
DTL	170.061909	0.9312368	0.24258363	3.91665928	8.9785E-05	0.01771049

Table S6. Capan-1 gene expression changes induced by ASC co-culture. Comparison is for FACS-sorted GFP+ cells: ASC / Capan-1 vs Capan-1 alone. To remove the background of mesenchymal genes (transmitted by exosomes or/and from occasional ASCs stuck to GFP+ Capan-1 during FACS), 3,764 genes differentially expressed by ASCs but not by Capan-1 cells (padj<0.05; log2FoldChange > 0) were removed from the list. Shown are top genes most upregulated by ASCs.

	baseMean	log2FoldChan	lfcSE	stat	pvalue	padj
OLFM4	1405.74584	0.97769819	0.20202353	1.22866842	0.21919615	0.99982974
LGALS4	94.0350961	0.91257996	0.20339452	2.33681733	0.01944868	0.51425694
CA9	21.0072619	0.78615893	0.18528227	1.264172	0.2061683	0.99982974
AC020978.6	57.1641941	0.73163932	0.20334785	1.63832762	0.10135337	0.90232573
LYZ	723.38622	0.71658144	0.19991307	0.77426138	0.43877624	0.99982974
NOXA1	46.0831666	0.70090519	0.20350669	0.80227036	0.42239658	0.99982974
IHH	42.7203122	0.68969464	0.19538023	0.89031889	0.37329468	0.99982974
GCNT3	1436.64188	0.68180251	0.1542433	1.97111357	0.04871089	0.74202453
USH1C	145.231121	0.67703657	0.19982735	1.51983163	0.1285533	0.94928106
SPINK1	574.682043	0.662423	0.19628994	0.75691967	0.44909799	0.99982974
CEACAM5	705.959457	0.65654734	0.19235372	1.12555239	0.26035503	0.99982974
CEACAM6	5356.60781	0.6302015	0.15985741	1.76963767	0.07678752	0.83574373
TFF1	513.927486	0.62761756	0.20243167	1.10744026	0.26810363	0.99982974
CKMT1A	119.848401	0.6086742	0.20338797	2.25494309	0.0241369	0.56598973
AC034236.1	49.3812426	0.60442846	0.19606703	2.65502833	0.00793017	0.3272347
KRT13	575.142883	0.6004594	0.20071435	0.3981911	0.69048933	0.99982974
VSIG2	88.1442488	0.59745307	0.1973416	1.24570341	0.2128733	0.99982974
SHH	255.31214	0.57915166	0.20314931	0.80163878	0.42276194	0.99982974
ADRA2A	25.1542561	0.57493024	0.19085904	0.47933661	0.63169918	0.99982974
ITGB4	2073.15623	0.57113292	0.1483095	2.57942431	0.00989651	0.36933698
MMP28	131.3994	0.57075441	0.19123485	0.99199605	0.32119945	0.99982974
CTSE	421.161874	0.57068075	0.16484783	2.81569944	0.00486712	0.24257037
ARL14	65.2646817	0.56973671	0.19772724	1.68657309	0.09168547	0.87970587
APOL1	870.698001	0.5691381	0.12663737	3.77603046	0.00015935	0.02201372
S100A5	16.2572448	0.56457626	0.16633059	1.12274625	0.26154528	0.99982974
FA2H	205.335639	0.56389119	0.17066432	2.26116937	0.02374877	0.56158977
NRG3	21.6560061	0.55587186	0.2013555	0.62683282	0.53076883	0.99982974
MSLN	106.781073	0.55478185	0.20337263	1.6137173	0.10658876	0.91430631
SEMA4B	1092.82927	0.54990453	0.15564844	2.90714577	0.00364743	0.20226551
CFTR	183.250597	0.53769543	0.20161777	0.3497325	0.72653946	0.99982974
DEFB1	84.1287865	0.53435729	0.19989477	1.77454875	0.07597243	0.83245289
OVOL2	16.9242179	0.5334697	0.190294	1.54357887	0.12269039	0.93883717
PPP1R1B	1157.32368	0.53180296	0.20002097	0.23700272	0.81265468	0.99982974
CASP14	18.9458065	0.52895168	0.15839575	0.67285681	0.50103839	0.99982974
MUC5B	4922.60918	0.52812613	0.17768387	0.78186257	0.43429535	0.99982974
ANXA10	510.315567	0.52535675	0.15763461	1.8870559	0.05915281	0.78401587
H3F3AP4	228.143344	0.52439458	0.18727696	2.96316113	0.00304497	0.18164868
SDHAF1	61.7705499	0.52012397	0.19546161	1.40572592	0.15980553	0.99422575
TMEM176A	45.0431148	0.5141276	0.20243915	0.95479535	0.3396812	0.99982974
AL031282.2	39.8089829	0.51085387	0.19042371	1.36803128	0.17130229	0.99982974
CAPG	389.996835	0.51016066	0.15084222	2.79459124	0.00519654	0.25337544
MGAM2	77.3833602	0.50946468	0.20342771	0.60111109	0.547766	0.99982974
TRIM52-AS1	14.9128195	0.50929444	0.19391099	2.57354841	0.01006616	0.37040163
PRSS21	103.653751	0.50918767	0.19187394	1.4649214	0.14294236	0.96905549
KALRN	82.961644	0.50808636	0.2019659	1.60824156	0.10778228	0.91705196
FAM83A	47.4536261	0.50670932	0.2005805	1.24610625	0.21272539	0.99982974
EPHA4	196.615882	0.50582035	0.18594396	1.78009168	0.07506096	0.82956844
TMTC4	82.5111743	0.4957354	0.20262726	1.95593708	0.05047257	0.75015562
EFNA2	25.2100533	0.49383995	0.1849558	0.74275573	0.45762958	0.99982974
DHFR2	69.8958488	0.49363118	0.19820279	1.59431217	0.11086613	0.92582086

https://doi.org/10.3390/cancers18020233

Table S7. Expression changes in Capan-1 cells upon *LINC01614* KO in co-cultured ASCs. Comparison is for GFP+ FACS-sorted cells from co-cultures ASC / Capan-1 vs *LINC01614*-KO ASC / Capan-1. To remove the background of mesenchymal genes (transmitted by exosomes or/and from occasional ASCs stuck to GFP+ Capan-1 during FACS), 3,726 genes differentially expressed by ASCs but not by Capan-1 cells (padj<0.05; log2FoldChange > 0) were removed from the list. Shown are top genes most reduced by *LINC01614* KO.

	baseMean	log2FoldChan	lfcSE	stat	pvalue	padj
OLFM4	1405.74584	1.65956207	0.3117189	1.76788143	0.07708073	0.41287624
NOXA1	46.0831666	1.28387434	0.29736126	3.52740017	0.00041966	0.02521483
IHH	42.7203122	1.24386055	0.32711943	2.60221677	0.00926233	0.14266483
PGC	27.1494994	1.16802266	0.28969499	1.54385136	0.12262435	0.50134419
ADRA2A	25.1542561	1.10040352	0.3312206	1.82032819	0.06870904	0.39273835
BPIFB1	306.16096	1.0616803	0.18645759	4.53275775	5.8219E-06	0.00141989
TMC4	264.131085	1.05562756	0.19665721	4.24296596	2.2058E-05	0.00343336
DMBT1	5800.37216	1.00833186	0.28645617	2.03438493	0.0419128	0.3116293
CEACAM6	5356.60781	1.00631079	0.1887919	2.67859741	0.00739312	0.12964614
NRG3	21.6560061	0.99345813	0.31466075	1.01972554	0.30785865	0.72521341
ONECUT3	70.4171961	0.96965635	0.27125476	1.76083862	0.07826572	0.41546123
EFNA2	25.2100533	0.95887776	0.33410618	2.98445972	0.0028408	0.07463771
GCNT3	1436.64188	0.94940251	0.18181263	2.2045022	0.02748905	0.25184138
AC034236.1	49.3812426	0.91243044	0.32579732	2.06036212	0.03936394	0.30195703
OVOL2	16.9242179	0.91142041	0.33223234	1.18214711	0.23714733	0.65466208
COLCA2	44.3346643	0.90205167	0.26639879	2.08992594	0.03662445	0.29064183
TMEM238	128.18594	0.89958419	0.27047277	2.49239836	0.01268836	0.16889075
ADGRB1	33.7193947	0.89891071	0.33209478	1.53971077	0.12363087	0.50210116
AC020978.6	57.1641941	0.86635384	0.30252088	-0.587941	0.55657192	0.8789174
MGAM2	77.3833602	0.86535001	0.30067815	0.99924644	0.31767532	0.73242005
CRIP1	338.177591	0.86045048	0.18424886	2.94496487	0.00322991	0.08077997
SSTR5-AS1	30.5128986	0.85893167	0.33317631	2.01126505	0.04429747	0.32148707
OAS1	270.8756	0.85604312	0.21743588	2.25411318	0.02418905	0.23433552
TMEM176A	45.0431148	0.84465912	0.30959795	0.84955182	0.39557431	0.78709575
OVOL1	113.253258	0.83513837	0.23137142	2.1195369	0.03404512	0.28010607
APOL1	870.698001	0.8288174	0.15159789	4.65666571	3.2137E-06	0.00095031
LYPD2	147.702276	0.81753008	0.20127013	2.76990096	0.00560733	0.11126869
UPK3A	17.3041979	0.81477235	0.32069147	3.24087493	0.00119163	0.0450402
CMTM8	53.0917592	0.81407818	0.26717133	2.56927852	0.01019105	0.14863757
FAM213B	342.96729	0.80421533	0.21528346	3.46528605	0.00052967	0.02896741
ELMO3	206.313799	0.79857097	0.20871853	2.77677831	0.00549006	0.10995959
UBXN10	49.7668589	0.79825398	0.3173706	1.83914944	0.06589321	0.38784049
PAK6	93.2921091	0.79672555	0.29089218	1.41904118	0.15588701	0.55147361
SP6	55.9375573	0.79563376	0.31601432	1.9143185	0.05557947	0.35973992
MSLN	106.781073	0.79409562	0.29444586	1.60308745	0.10891535	0.47915764
ZFP36	767.365841	0.78832177	0.15538718	4.84899454	1.2409E-06	0.00049247
RNA5-8SN3	12759.8746	0.7870769	0.2750248	2.36190742	0.01818118	0.20146246
RNA5-8S4	12759.8746	0.7870769	0.2750248	2.36190742	0.01818118	0.20146246
RNA5-8SN4	12759.8746	0.7870769	0.2750248	2.36190742	0.01818118	0.20146246
RNA5-8SN2	12759.8746	0.7870769	0.2750248	2.36190742	0.01818118	0.20146246
RNA5-8S5	12759.8746	0.7870769	0.2750248	2.36190742	0.01818118	0.20146246
RNA5-8SN5.1	12759.8746	0.7870769	0.2750248	2.36190742	0.01818118	0.20146246
FAM83A	47.4536261	0.78578701	0.31703754	0.85896114	0.39036195	0.78277595
ITGB4	2073.15623	0.78426427	0.17481753	2.8020737	0.00507753	0.10504004
CAMK2N1	524.796429	0.78418391	0.14416818	5.27618846	1.319E-07	6.8592E-05
KLK1	21.9940748	0.7828979	0.33425351	1.37188103	0.17010048	0.57172091
SEMA4G	210.336836	0.78274307	0.22850887	2.45515077	0.01408255	0.17861981
MPND	104.809406	0.78169485	0.21046852	4.67489909	2.941E-06	0.00095031

Table S8. Expression changes in Capan-1 cells upon *SFRP4* KO in co-cultured ASCs. Comparison is for GFP+ FACS-sorted cells from co-cultures ASC / Capan-1 vs *SFRP4*-KO ASC / Capan-1. To remove the background of mesenchymal genes (transmitted by exosomes or/and from occasional ASCs stuck to GFP+ Capan-1 during FACS), 3,967 genes differentially expressed by ASCs but not by Capan-1 cells (padj<0.05; log2FoldChange > 0) were removed from the list. Shown are top genes most reduced by *SFRP4* KO.

	baseMean	log2FoldChan	lfcSE	stat	pvalue	padj
OLFM4	1405.74584	1.0633295	0.22883941	1.33139438	0.18305928	0.6105465
NOXA1	46.0831666	0.86148125	0.22774652	2.06232324	0.03917697	0.31424324
IHH	42.7203122	0.80133633	0.22505794	1.46317693	0.14341897	0.55415601
AGPAT2	320.249735	0.79969079	0.13709506	5.69117207	1.2617E-08	1.3247E-05
MRPL23	250.258551	0.78621923	0.18846867	4.92155494	8.5859E-07	0.00024585
PGC	27.1494994	0.75614749	0.22619316	1.404115	0.16028461	0.5810832
TP53INP2	198.514907	0.74001141	0.15818747	5.90690927	3.4859E-09	4.6705E-06
LPCAT2	278.334002	0.71627785	0.15495473	3.93277247	8.3972E-05	0.00870046
PPP1R1B	1157.32368	0.70018004	0.21970562	1.59408128	0.11091783	0.49756918
KRT13	575.142883	0.69590019	0.2284352	0.90631052	0.36477152	0.78427055
RPL23AP42	3379.67435	0.69575788	0.13854342	5.04014385	4.6518E-07	0.00017401
NRG3	21.6560061	0.69523912	0.22857914	1.9857761	0.04705819	0.34406659
ADRA2A	25.1542561	0.69132804	0.22139514	1.24787513	0.21207679	0.64665971
Telomerase-ve	510.078236	0.68880561	0.16869249	4.16390348	3.1285E-05	0.00407199
RPL18AP3	268.088643	0.68623865	0.16860194	4.80185818	1.572E-06	0.00041962
SDHAF1	61.7705499	0.68569109	0.21235147	3.0794624	0.00207375	0.06467211
AC034236.1	49.3812426	0.67473473	0.2255152	2.29497615	0.02173449	0.23528046
PLPPR2	437.602209	0.66349626	0.16497754	5.73453632	9.7779E-09	1.0999E-05
SERINC2	751.573915	0.66286496	0.11637836	5.53331631	3.1423E-08	2.1434E-05
ITGB4	2073.15623	0.65816387	0.15410695	3.30342021	0.00095513	0.04098737
S100A5	16.2572448	0.6573227	0.19784746	2.66202479	0.00776722	0.13766521
IL10RB	164.508922	0.64783326	0.19838865	3.51361005	0.00044206	0.02629656
AC090498.1	628.255622	0.64609121	0.1655472	3.73681098	0.00018637	0.01467562
FA2H	205.335639	0.64206956	0.18027052	2.93317311	0.00335517	0.08468034
CENPS	51.5416667	0.6396181	0.22641788	4.38508824	1.1594E-05	0.00181147
MSLN	106.781073	0.63759963	0.22716061	2.78024102	0.00543186	0.11159656
MIEN1	315.09615	0.63471925	0.16067352	3.47424829	0.00051229	0.02830879
RNA5-8SN3	12759.8746	0.63440482	0.22174843	3.22886543	0.00124282	0.04671415
RNA5-8S4	12759.8746	0.63440482	0.22174843	3.22886543	0.00124282	0.04671415
RNA5-8SN4	12759.8746	0.63440482	0.22174843	3.22886543	0.00124282	0.04671415
RNA5-8SN2	12759.8746	0.63440482	0.22174843	3.22886543	0.00124282	0.04671415
RNA5-8S5	12759.8746	0.63440482	0.22174843	3.22886543	0.00124282	0.04671415
RNA5-8SN1	12759.8746	0.63440482	0.22174843	3.22886543	0.00124282	0.04671415
RNA5-8SN5	12759.8746	0.63440482	0.22174843	3.22886543	0.00124282	0.04671415
RNA5-8SN5.1	12759.8746	0.63440482	0.22174843	3.22886543	0.00124282	0.04671415
KIAA1161	189.565783	0.63431374	0.16664806	3.81629117	0.00013547	0.01191932
TMEM238	128.18594	0.63294845	0.22013185	2.63958308	0.00830081	0.14203093
C19orf33	937.710032	0.63042478	0.14156297	4.06973333	4.7067E-05	0.00553178
C8orf82	136.156214	0.63025227	0.17596355	4.08518828	4.4041E-05	0.00533541
MLPH	720.407149	0.62844294	0.12405479	4.45542983	8.3725E-06	0.00144152
RPL18A	4187.56207	0.61871557	0.10859077	5.61916386	1.9188E-08	1.3736E-05
HSD3B7	174.373389	0.61693289	0.16750059	4.68783927	2.761E-06	0.0006212
ALPP	1104.98819	0.61608724	0.16877141	3.06642318	0.00216636	0.06650697
PMS2P1	115.079809	0.61430785	0.21558381	3.04566338	0.00232168	0.06815975
ATP6V0E1	627.551819	0.61137842	0.13002953	5.62873895	1.8153E-08	1.3736E-05
TMC4	264.131085	0.59646461	0.17472394	2.12207771	0.03383121	0.2897269
GCNT3	1436.64188	0.59450608	0.16088118	1.45844909	0.1447168	0.55610985
ARL14	65.2646817	0.59349578	0.21584209	2.05945906	0.03945028	0.31490243
AC020978.6	57.1641941	0.58933742	0.22854059	-0.1468305	0.88326583	0.97600762
CLTB	1356.76157	0.58913736	0.13320005	5.08992689	3.582E-07	0.00014103

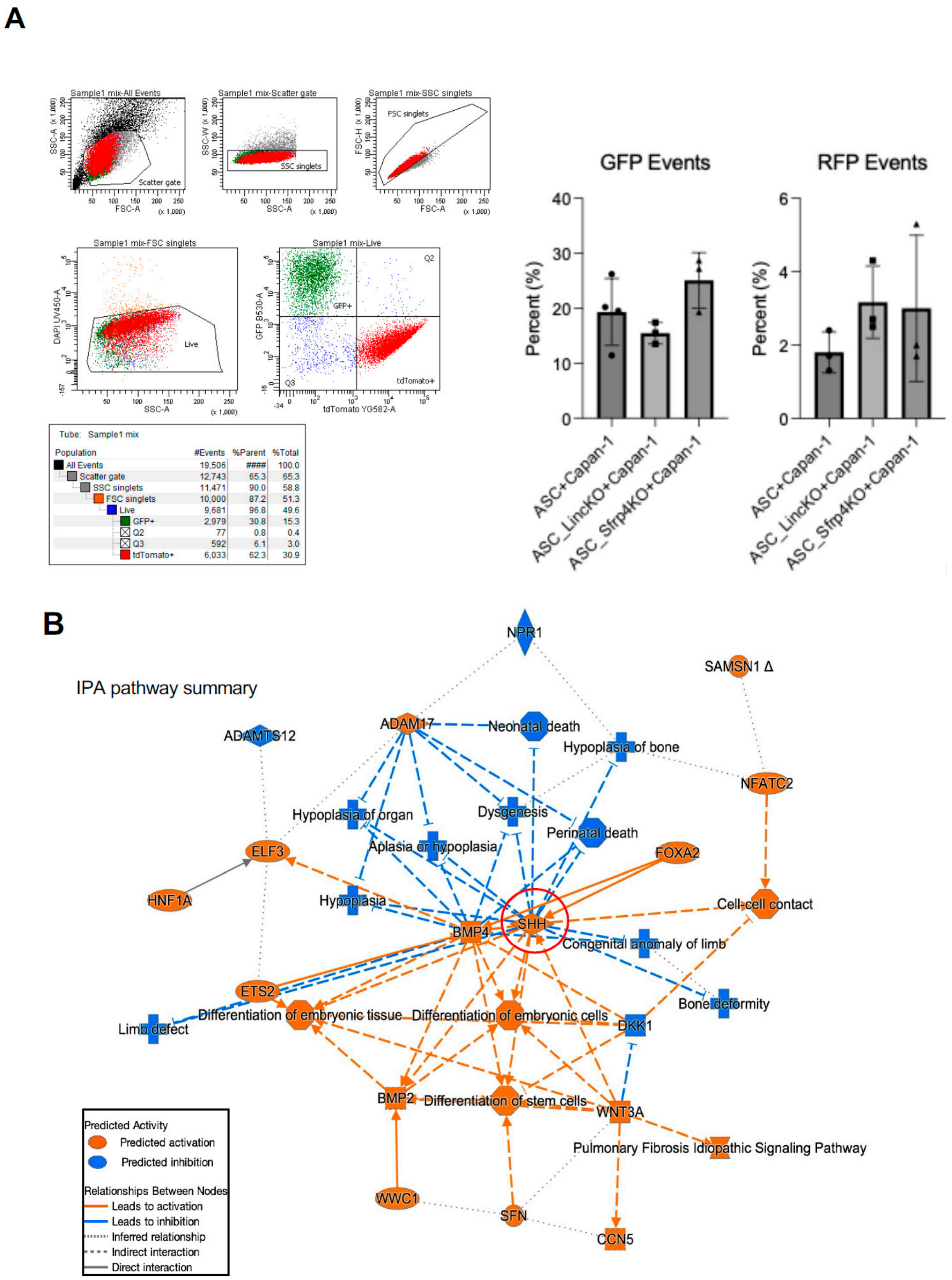

Figure S1. The effect of PDAC cell co-culture on ASCs. ASCs expressing RFP were cultured with or without Capan-1 cells expressing GFP, co-seeded at a 1:1 ratio. **A** After 7 days of co-culture, cells were FACS-separated and quantified (graphs). Plotted are mean values from three independent co-culture experiments and sorts. **B** Top pathway changes induced in ASC by Capan-1 co-culture identified in RNA-seq data by IPA. Activation of SHH signaling is highlighted.

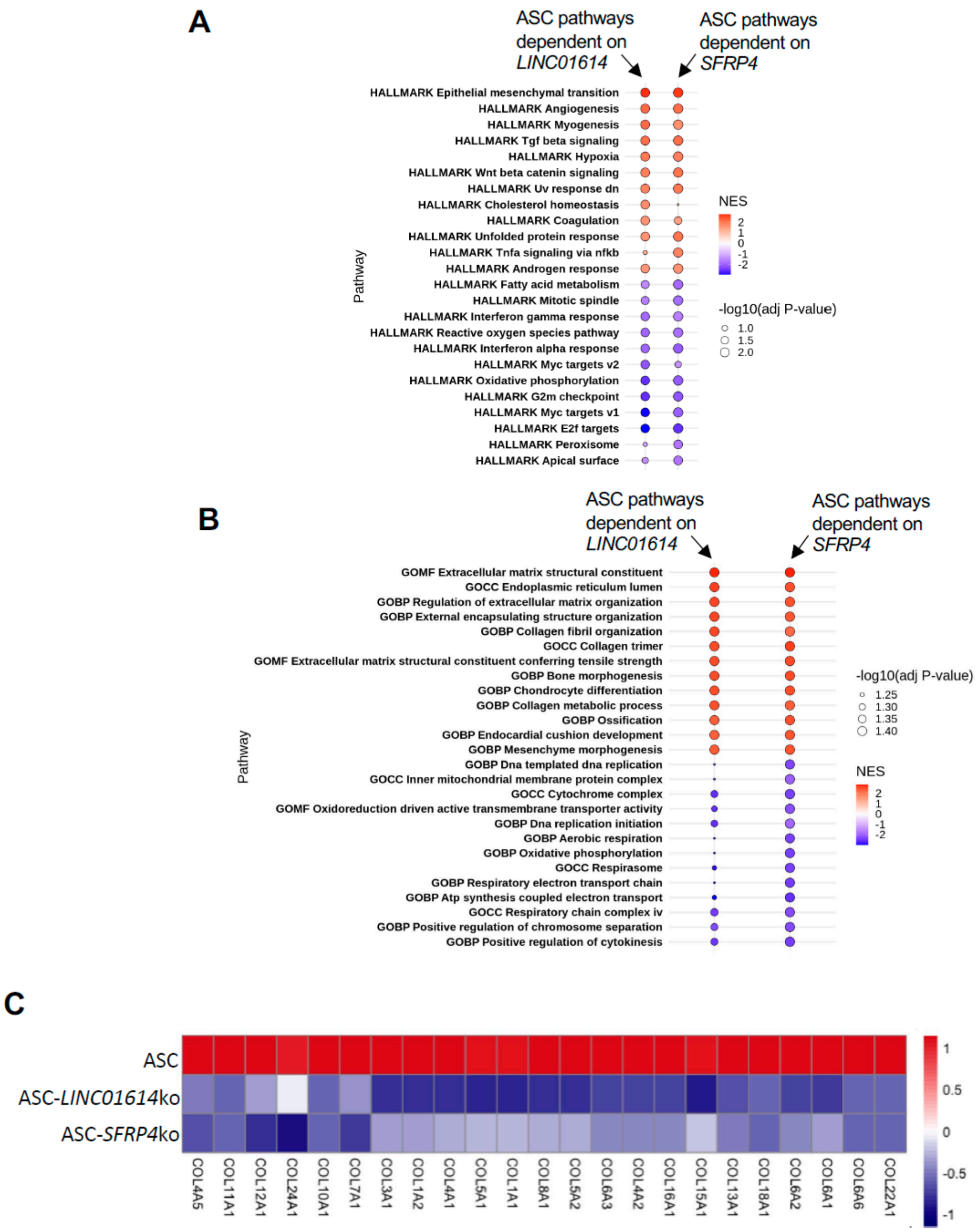

Figure S2. The effect of SFRP4 and LINC01614 knockout on ASC gene expression in Capan-1 co-culture analyzed by GSEA. A Plot of normalized enrichment scores (NES) and adjusted *P* values for the combined set of the top ten most upregulated and top ten most downregulated Hallmark gene sets from each indicated comparison. The first column shows ASC + Capan-1 co-culture versus ASC-LINC01614 knockout + Capan-1 co-culture, while the second column shows ASC + Capan-1 co-culture versus ASC-SFRP4 knockout + Capan-1 co-culture. The color scale represents the NES calculated by GSEA, and the circle size indicates significance level, measured as −log10(adjusted p value). **B** Same as panel A, showing the top ten most upregulated and top ten most downregulated Gene Ontology (GO) terms instead of Hallmark gene sets. **C** Expression of genes identified by Gene Set Enrichment Analysis (GSEA) for the GO molecular function term “GOMF extracellular matrix structural constituent conferring tensile strength” compared among parental, LINC01614-ko, and SFRP4-ko ASCs after co-culture with Capan-1. The heatmap shows gene-wise z-score-scaled average expression per condition. The scale bar indicates relative gene expression from high (red) to low (blue).

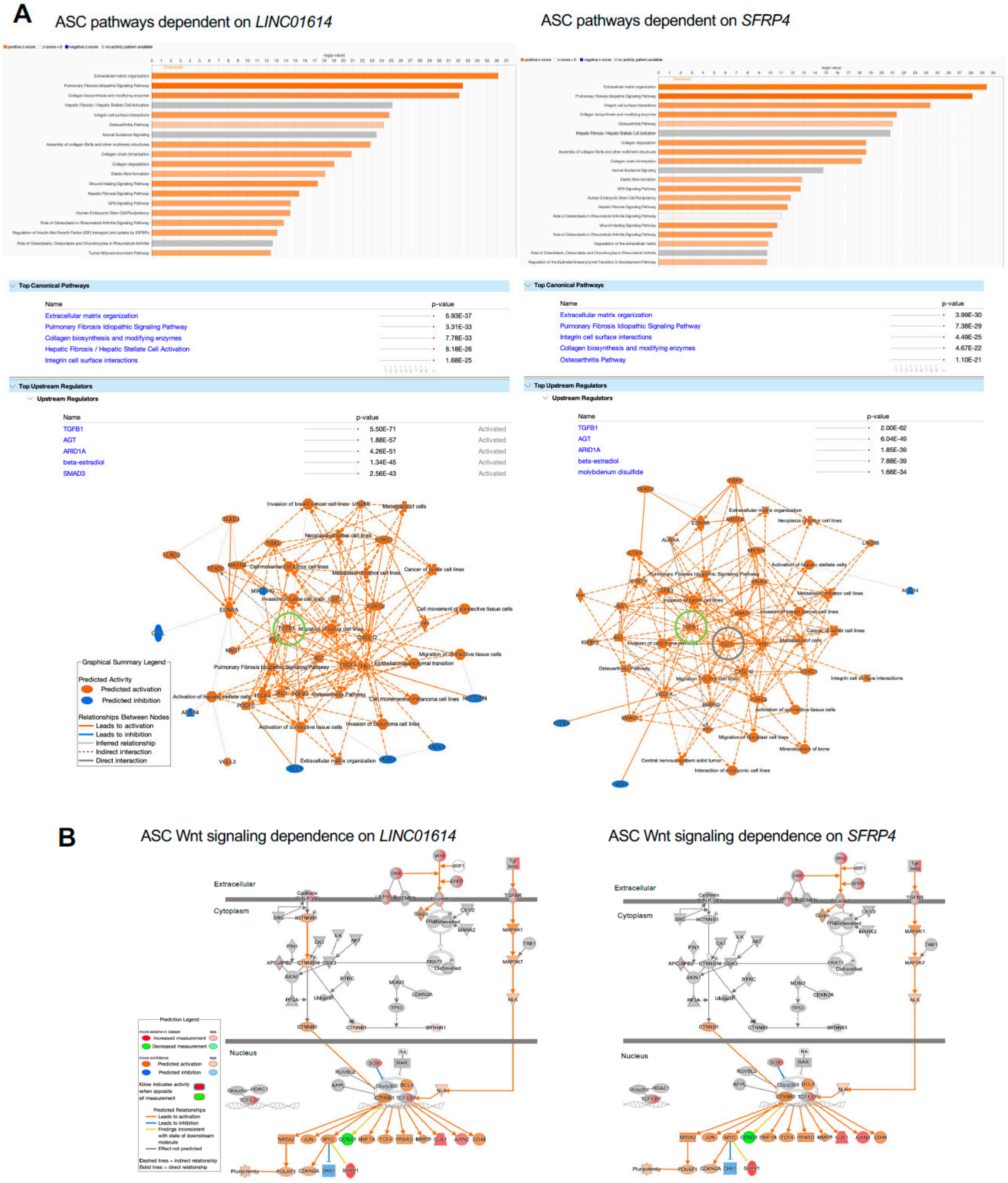

Figure S3. The effect of *SFRP4* and *LINC01614* knockout on ASC gene expression in Capan-1 co-culture analyzed by IPA. **A** Top cellular processes and canonical pathways downregulated following *LINC01614* ko or *SFRP4* ko in Capan-1 co-culture. TGFB1 and GLI1 signaling being centered on are highlighted. **B** Signaling focused on TGFβ and Wnt pathways, downregulated by *LINC01614* ko and *SFRP4* ko in Capan-1 co-culture.

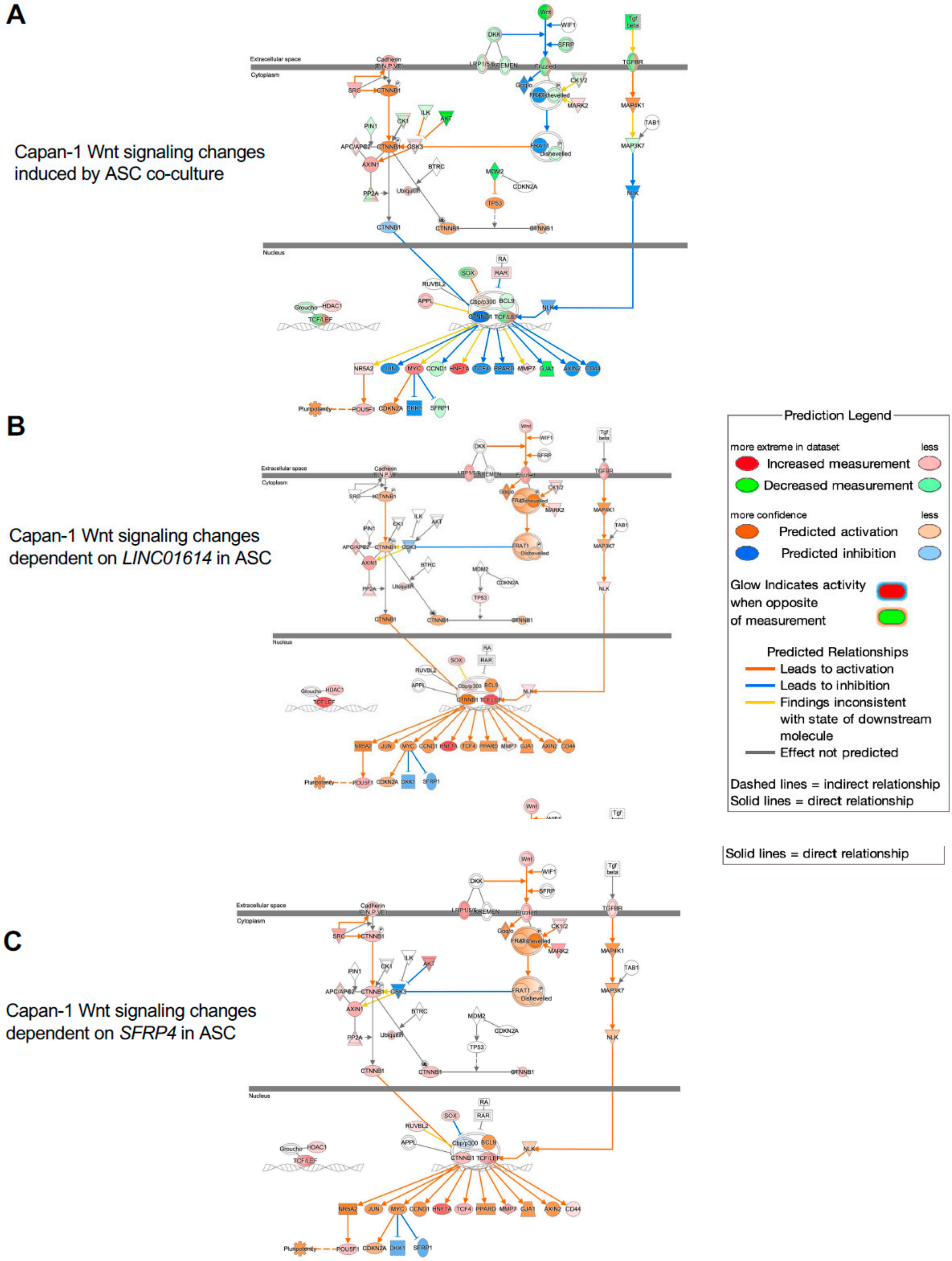

Figure S4. The effect of ASC, *SFRP4* ko in ASC, and *LINC01614* ko in ASCs on cancer cells. Total RNA expression from Capan-1 cells co-culture with parental ASCs were compared with those co-cultured with *LINC01614*-ko ASC or *SFRP4*-ko ASC, and the resulting differential expression results were analyzed by IPA with a focus on the TGFβ / Wnt signaling pathways. **A** Capan-1 vs Capan-1 + parental ASCs; **B** Capan-1 + parental ASCs vs Capan-1 + *LINC01614*-ko ASCs; **C** Capan-1 + parental ASCs vs Capan-1 + *SFRP4*-ko ASCs.

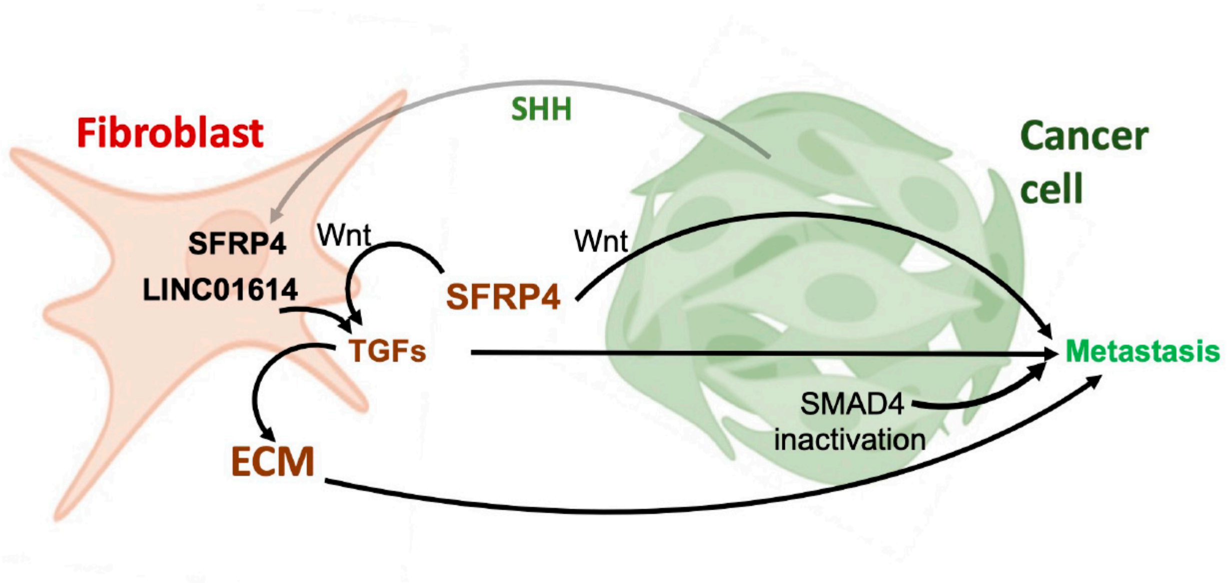

Figure S5. The model of ASC-CAF cross-talk with PDAC cells. Upregulation of *LINC01614* and *SFRP4* by cancer cells induces Wnt signaling and the expression of TGFs and ECM in CAFs. TGFs and SFRP4 secreted from CAFs promote Wnt and TGF signaling in cancer cells, which synergize with desmoplastic changes, promote PDAC cell proliferation, survival, and migration, which collectively enable metastasis. Inactivation of SMAD4 short-circuits cancer cells to become more metastatic irrespective of SFRP4 signaling.
